# Urbanization and prevalence of type 2 diabetes in Southern Asia: A systematic analysis

**DOI:** 10.7189/jogh.04.010404

**Published:** 2014-06

**Authors:** Arsalan Cheema, Davies Adeloye, Simrita Sidhu, Devi Sridhar, Kit Yee Chan

**Affiliations:** Centre for Population Health Sciences and the World Health Organization's Collaboration Centre for Population Health Research and Training, The University of Edinburgh Medical School, Edinburgh, Scotland, UK

## Abstract

**Background:**

Diabetes mellitus is one of the diseases considered to be the main constituents of the global non*–*communicable disease (NCD) pandemic. Despite the large impact that NCDs are predicted to have, particularly in developing countries, estimates of disease burden are sparse and inconsistent. This systematic review transparently estimates prevalence of type 2 diabetes mellitus in Southern Asia, its association with urbanization and provides insight into the policy challenges facing the region.

**Methods:**

The databases Medline and PubMed were searched for population*–*based studies providing estimates of diabetes prevalence in the Southern Asia region. Studies using WHO diagnostic criteria of fasting plasma glucose (FPG) ≥7.0mmol/L and/or 2h*–*plasma glucose (2hPG) ≥11.1mmol/L were included. Data from eligible studies was extracted into bubble graphs, and trend lines were applied to UNPD figures to estimate age*–*specific prevalence in the regional population. Estimates specific to sex, area of residency, and diagnostic method were compared and trends analysed.

**Results:**

A total of 151 age*–*specific prevalence estimates were extracted from 39 studies. Diabetes prevalence was estimated to be 7.47% for 2005 and 7.60% for 2010. Prevalence was strongly associated with increased age, male gender and urban residency (*P* < 0.001).

**Conclusion:**

Diabetes prevalence in Southern Asia is high and predicted to increase in the future as life expectancy rises and the region continues to urbanise. Countries in this region need to improve NCD surveillance and monitoring so policies can be informed with the best evidence. Programs for prevention need to be put in place, and health system capacity and access needs to be assessed and increased to deal with the predicted rise in NCD prevalence.

In recent years the issue of non*–*communicable diseases (NCDs) has been identified as a pressing concern that has come to the forefront of international policy discussion. NCDs are the leading causes of death and disability worldwide [1]. It was estimated that 33 million deaths in 2008 occurred from NCDs, accounting for almost two*–*thirds of all deaths for that year [2]. In addition, estimates suggest that these may increase further to a projected 52 million deaths by 2030, nearly five times as many deaths as projected for communicable diseases [3]. Type 2 diabetes mellitus (DM), along with cardiovascular disease, cancers, and chronic respiratory diseases, are considered four primary constituent diseases of the global NCD pandemic [3,4]. Importantly, the main risk factors for these diseases are modifiable and these diseases are heavily influenced by lifestyle and behaviour [5]. Shared risk factors between these diseases – such as eating an unhealthy diet high in saturated fat and sugar, a lack of physical activity, and tobacco smoking – account for over two*–*thirds of new NCD cases and increase the risk of exacerbations in those who already have these diseases [5]. These risk factors and resulting diseases are not limited to high*–*income countries – a disproportionate NCD burden is borne by developing countries. Over 80% of diabetes and cardiovascular deaths worldwide occur in low*–* and middle*–*income countries (LMICs), and many of the risk factors for these NCDs are associated with the country development process through globalisation and urbanisation [3]. The prevalence of the common NCDs increases with advancing age, so as life expectancy in LMICs increases the burden of NCDs is also expected to rise.

Non*–*communicable diseases are more likely to affect people who are socioeconomically disadvantaged, furthering health inequalities [6]. This can be due to contextual factors relating to the society and place in which people live in addition to behavioural factors. In LMICs, diabetes and its risk factors are associated with lower education levels [3]. The higher burden of NCDs poses additional problems for populations of developing countries that have lower levels of educational achievement and income. Limited health care capacities and lack of social protection for large parts of the population mean that treatment and support for NCDs is often unavailable or catastrophically expensive [5,7]. In addition, NCDs have significant socioeconomic effects. Nearly a third of NCD deaths in LMICs occur below the age of 60 [1]. These deaths at economically and socially productive ages have much wider consequences for these developing countries, with the loss of productivity and health system expenditure becoming major barriers for national economic development and progress [5,8]. On individual or household levels, the sustained nature of NCDs and resulting disabilities can lead to difficulties in working or seeking employment. Additionally, the long*–*term care that NCDs require and the high cost of health care in many developing countries have major impacts on household income, potentially leading to vicious cycles of poverty and illness [5]. The overall economic cost of NCDs cannot be understated: in India in 2004–2005, NCD health care expenditure and total income lost due to these diseases was estimated to amount to 1% of its massive economy [3].

Despite the serious implications of the global NCD burden, it is only recently that determined policy action has been seen. The UN High*–*Level Meeting on NCDs in 2011 led the way for an international response, providing guidance on how to integrate NCD prevention and control across sectors and at all levels of government [1,3]. Furthermore, monitoring and surveillance capabilities of several high*–*burden countries have shown an increased capacity in recent years [2]. However, many of the recommended changes – such as health care system reform towards sustainable universal care, and integration of NCD prevention into multi*–*sectoral responses – may take several years to implement, particularly in the LMICs that bear the brunt of the global NCD burden. Meanwhile it is essential that these regions have reliable estimates of NCD burden to inform policy decisions with relevant evidence and help set appropriate health care and research priorities [9]. Transparent, up*–*to*–*date estimates of NCD burden allow monitoring of the diseases as well as evaluation of current policies, and are vital tools for planning policies and interventions to tackle the global NCD pandemic. This paper will attempt to address part of this need by carrying out a systematic literature review to estimate the prevalence of type 2 DM in Southern Asia.

**Box 1** briefly reviews approaches to diagnosis and known risk factors for type 2 diabetes [10-18]. **Table 1** displays current WHO diagnostic criteria for venous plasma for fasting plasma glucose (FPG) and oral glucose tolerance test (OGTT). Diagnosis can be made through the use of either test alone or together. Specific values for capillary measurements and whole blood have been provided in previous WHO publications as well [12]. In terms of geographic focus of this study, the UN's Southern Asia region is comprised of nine countries [19]. General information regarding each country is given in **Table 2**, sourced from the World Bank online database [20]. The majority of Southern Asian countries are low or low*–*middle income countries [20]. The total population of the Southern Asia region comprises approximately 25% of the world total population [20]. India was estimated to have the highest number of diabetic adults in 2000 [21] and 2010 [22], and both these studies predicted it would continue to have the highest number of diabetic adults by 2030. Pakistan and Bangladesh were both estimated to be in the top ten as well. As such, an estimate of the diabetes prevalence for this region would provide a major insight into the global picture of diabetes burden.

Box 1 **Type 2 diabetes – diagnosis and risk factors**

Type 2 diabetes mellitus (DM) is a metabolic disease characterised by persistent hyperglycaemia and disturbed carbohydrate, protein, and fat metabolism. It may present with combinations of typical symptoms such as polydipsia (increased thirst), polyphagia (excessive hunger), polyuria (increased passage of urine), glycosuria (glucose in urine), lethargy, and weight loss. These symptoms reflect the underlying DM pathophysiology of peripheral insulin resistance combined with inadequate pancreatic insulin secretion [10]. Many diabetic patients may be asymptomatic but in the long term uncontrolled hyperglycaemia can lead to severe complications such as diabetic retinopathy, neuropathy and nephropathy. Type 2 DM can be diagnosed through biochemical measurements even if there are no presenting symptoms [11].

Under World Health Organisation (WHO) guidelines, there are currently two main diagnostic tests used to diagnose DM – the Fasting Plasma Glucose (FPG) test and the Oral Glucose Tolerance Test (OGTT) [11]. FPG involves measuring the level of glucose in a fasting (≥8 hours without food) patient’s blood, often after an overnight fast. OGTT is also carried out on fasting patients and involves measurement of baseline blood glucose, followed by ingestion of 75g anhydrous glucose, and a subsequent blood glucose measurement after two hours to determine the efficacy with which glucose has been eliminated from the patient’s blood [12]. Although WHO has also recently advocated the measurement of glycatedhaemoglobin (HbA1_C_) for diagnostic purposes [13], the stringent quality assurance tests required for its effective usage have limited its use in epidemiological studies to date. Additional notes on these diagnostic methods are provided in **Online Supplementary Document(Online Supplementary Document)**.

The aetiology of type 2 DM is complex and likely involves a host of different factors, many of which are not fully understood. Common risk factors in the general population include older age, being overweight or obese, hypertension, leading an inactive lifestyle, smoking, and consuming an energy*–*dense diet [14,15]. Several of these risk factors may be considered ‘lifestyle’ factors that are potentially modifiable. However, a strong genetic component is also implicated in Type 2 DM, with relatives of diabetics at increased risk of developing it themselves, and certain ethnic populations believed to have increased susceptibility to diabetes [16,17]. South Asians in particular have been found to possess adverse body fat patterning that that may predispose to insulin resistance [18], and have higher diabetes risk than Caucasians with equivalent body mass indices (BMI) [16]. This non*–*modifiable genetic susceptibility for South Asians means it is of even greater importance that policies address modifiable risk factors in order to tackle burgeoning diabetes prevalence in the region.

**Table 1:** World Health Organization's 2006 diagnostic criteria for type 2 diabetes

Fasting plasma glucose (FPG) **≥7.0mmol/L (126mg/dL** and / or 2*–*hour plasma glucose (OGTT 2hPG) **≥11.1mmol/L (200mg/dL)**

FPG –fasting plasma glucose, OGTT –oral glucose tolerance test.

**Table 2:** Southern Asia countries – selected characteristics

Country	Population, ×1000 (2011)	Life expectancy at birth (2010)	GNI per capita (US$) (2011)	GDP US$ growth, % (2011)	Total expenditure on health (% GDP) (2011)	Physicians per 1000 population (2011)
Afghanistan	35 320	48	1140	+6%	9.6	0.210
Bangladesh	150 394	69	940	+7%	3.7	0.295
Bhutan	738	67	5570	+6%	4.1	0.023
India	1 241 492	65	3590	+7%	3.9	0.649
Iran	74 799	73	11 420†	+2%†	5.8*	0.890
Maldives	320	77	7430	+7%	8.5	1.595
Nepal	30 486	68	1260	+4%	5.4	0.210†
Pakistan	176 745	65	2870	+3%	2.5	0.813
Sri Lanka	20 869	75	5520	+8%	3.4	0.492

GNI – gross national income, GDP – gross domestic product

*Data from 2009, most recent available data but likely inaccurate following punitive economic sanctions.

†Data from 2004.

This paper aims: (i) to contribute to the evidence base on type 2 diabetes mellitus in Southern Asia by systematically reviewing the relevant literature; (ii) to compare the prevalence estimates provided through different methods of diagnosing type 2 diabetes mellitus; (iii) to provide an assessment of the role of urbanization on the burden of Type 2 diabetes mellitus in the UN Southern Asia region based on the best available evidence; and (iv) to discuss the significance of the regional estimate and the implications it may have on public health policy.

## METHODS

A systematic literature search of published studies providing population*–*based prevalence estimates of type 2 diabetes mellitus in Southern Asia was carried out. The online databases Medline and PubMed were searched, using the OVID search form for the Medline database and the default search engine for PubMed. Search terms for Medline and PubMed are given in **Table 3** and **Table 4** respectively. Both Medical Subject Headings (MeSH terms) and keywords were used for the Medline search. The Medline search was more focused due to OVID’s Advanced Search feature, while the PubMed search was left broader in order to pick up a larger selection of studies. The final searches were carried out on 13 February 2013. **Box 2** shows inclusion criteria, exclusion criteria and quality evaluation criteria.

**Table 3:** OVID Medline search terms

Search terms		No. of studies
1	Diabetes Mellitus, type 2/	74 709
2	“adult*–*onset diabetes”.tw	358
3	(diabetes adj2 type 2).tw	54 707
4	“non*–*insulin dependent diabetes”.tw	8297
5	NIDDM.tw	67 32
6	Diabetes Mellitus/ or Diabetes Mellitus, type 2/	155 830
**7**	**1 OR 2 OR 3 OR 4 OR 5 OR 6**	**173 525**
8	exp morbidity/ or exp mortality/	563 526
9	incidence.tw	464 724
10	(prevalen* or mortality or epidemiol*).tw	961 509
11	Epidemiology/	11 218
12	“cost of illness”/	15 625
13	(burden adj2 diseas*).tw	7633
**14**	**8 OR 9 OR 10 OR 11 OR 12 OR 13**	**1 586 465**
15	Bangladesh/ or Bhutan/ or India/ or Afghanistan/ or Iran/ or Nepal/ or Pakistan/ or “Sri Lanka”/	102 952
16	Indian Ocean Islands/	568
17	(afghan* or bangladesh* or bengal* or bhutan* or iran* or india* or nepal* or pakistan* or maldiv* or srilanka *).tw	122 321
**18**	**15 OR 16 OR 17**	**161 040**
**19**	**7 AND 14 AND 18**	**1896**
**20**	Limit 19 to (humans and yr = ”1980*–*Current”)	**1754** results

**Table 4:** PubMed search terms

Search terms	No. studies
Diabetes AND (Afghanistan OR Bangladesh OR Bhutan OR India OR Iran OR Maldives OR Nepal OR Pakistan OR Sri Lanka) AND (Epidemiology OR Incidence OR Prevalence OR Mortality)	**3899** results

Box 2 **Literature search: Inclusion criteria, exclusion criteria and quality evaluation criteria**

**Inclusion criteria:**

• Population*–* or community*–*based study in a Southern Asian country providing prevalence estimates of type 2 diabetes mellitus based on primary data.

• All published study designs and all languages.

• Studies post*–*1980 with ≥200 participants.

• Studies looking at adults (≥20 years).

• Studies diagnosing diabetes through biochemical measurements.

**Exclusion criteria:**

• Studies investigating other forms of diabetes, such as gestational diabetes or diabetes insipidus.

• Hospital*–* or clinic*–*based studies.

• Studies diagnosing diabetes through self*–*reported questionnaires or symptoms only.

• Study populations specifically predisposed to diabetes, such as relatives of known diabetics.

• Studies investigating prevalence of complications in a diabetic cohort without commenting on actual prevalence of diabetes in area or community.

**Quality evaluation criteria:**

• Diabetes diagnosed through fasting plasma glucose (FPG) after ≥8 hours fasting, and/or oral glucose tolerance test (OGTT) two hours after ingestion of 75g anhydrous glucose or equivalent.

• Appropriate diagnostic criteria for diabetes – most recent WHO recommendations of FPG ≥7.0mmol/L and/or 2hPG ≥11.1mmol/L for venous plasma, or equivalent for other sample types. Stated whether blood samples were venous or capillary, and whether whole blood or plasma was analysed.

• Clearly defined population recruited through representative sampling methods.

• Description of how known diabetics were accounted for.

### Study selection

**Figure 1:**
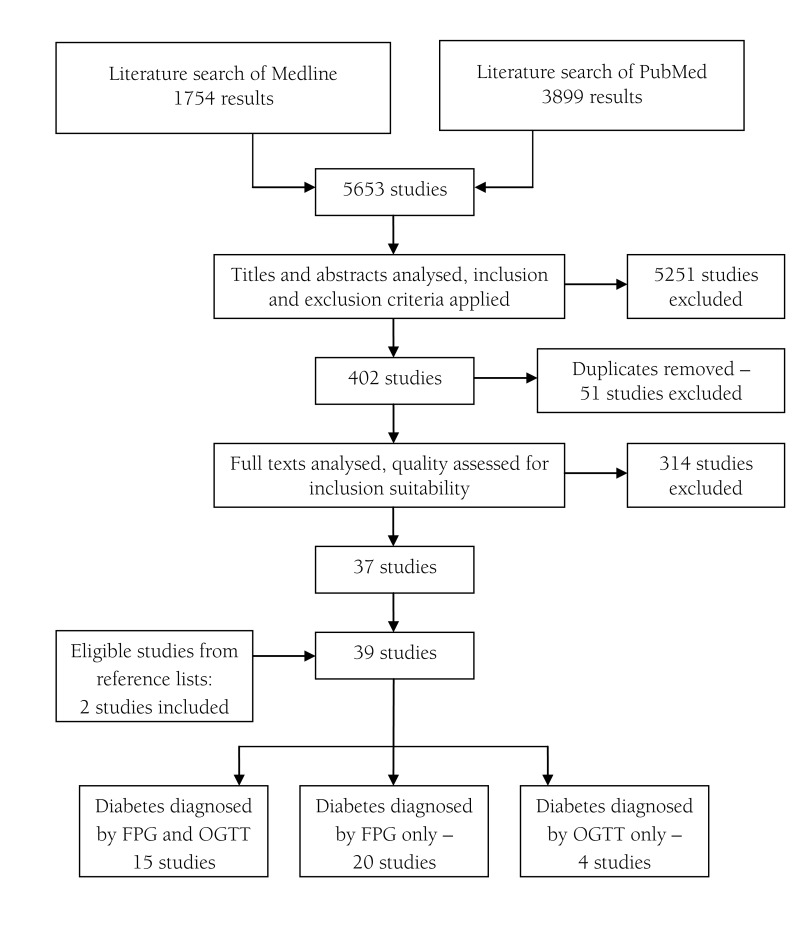
Study selection process. FPG –fasting plasma glucose, OGTT – oral glucose tolerance test.

The literature search of online databases resulted in a total of 5653 studies: 1754 from Medline and 3899 from PubMed. After initial analysis of titles and abstracts, 402 studies were selected that matched inclusion and exclusion criteria. 51 duplicate studies were removed and full texts of the remaining 351 studies were further analysed and quality assessed. 39 studies were included in the final analysis, including 2 papers identified through reference lists of other assessed studies. A visual summary of the study selection process is presented in **Figure 1**.

### Data extraction

Titles and abstracts of all studies obtained through the database searches were evaluated. Inclusion and exclusion criteria were applied. Basic details of all studies such as title, authors, country, study year, year of publication, and sample size were extracted into an Excel spreadsheet for ease of full text evaluation. After initial extraction, full texts of the studies were analysed and assessed for quality criteria. Quality assessment information is presented in **Online Supplementary Document(Online Supplementary Document)**. Studies for which full text was not available were requested through inter*–*library loans. Duplicate studies were identified through study locations and matching sample sizes, and were removed. In addition, the reference lists of the selected studies were examined for relevant papers not captured by the literature search. These new studies were subsequently evaluated and added to the spreadsheet.

Another Excel spreadsheet was created for eligible studies selected through full text analysis. All the above data was extracted in addition to data on method of diagnosis; diagnostic criteria; specific location of the study; whether the study described the surveyed area as urban, rural, mixed or none; age range of participants and mean age if provided; diabetes prevalence in sample; and sex*–*specific sample size, mean age, and diabetes prevalence. Many studies looked at several different cohorts in various areas, often for purpose of comparison. These multiple cohorts were recorded separately so that individual sample characteristics could be differentiated. Three separate sheets were created for studies depending on their method of diagnosing new diabetes: one for studies that diagnosed diabetes on the basis of both FPG and OGTT results; one for studies using only FPG results; and one for studies using only OGTT results. These spreadsheets were the basis for prevalence estimation.

### Data analysis

To allow for comparison between studies, all reported prevalence estimates were converted to prevalence/1000 population through the equation:

Prevalence = Number of diabetes cases ×1000 / Sample size

During data modelling, the mean age, sample size, and age*–*specific prevalence estimates (per 1000 population) of all selected studies were used to create bubble graphs representing the data. If this information was missing for particular cohorts, it was calculated from the data that was available, as detailed in **Online Supplementary Document(Online Supplementary Document)**. Several bubble graphs were created: for overall prevalence, sex*–*specific prevalence, urban/rural prevalence, and prevalence for specific diagnostic methods.

In order to calculate population prevalence estimates for the region, trend lines with the power function were computed from the graphs to represent the relationship between age and prevalence for the selected data set. These were chosen because they had the highest r*–*squared (R^2^) values for these graphs and therefore accounted for the highest fraction of variance in the data. Statistical significance (p*–*values) of differences observed through any comparisons was derived directly from the model. The resulting equations for overall combined prevalence, total male prevalence and total female prevalence were applied to 2005 UNPD population estimates, the closest to the median study year of 2006 [23]. Prevalence results were multiplied by population figures for each age group, thereby giving an estimate of the total number of expected diabetes cases for each one. Totalling these up and taking a percentage of the total adult population allowed calculation of overall diabetes prevalence, for each sex separately and combined.

### Study characteristics

**Figure 2:**
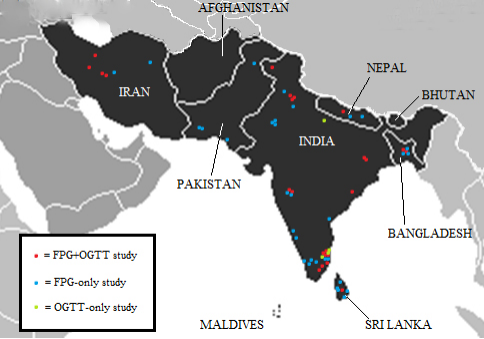
Approximate geographic locations of the study cohorts. FPG – fasting plasma glucose, OGTT – oral glucose tolerance test.

Of the 39 studies included in the analysis, 15 studies diagnosed diabetes using both FPG and OGTT methods, 20 solely using FPG, and 4 studies using OGTT only. Several studies looked at more than one cohort when estimating diabetes prevalence, often for purposes of comparison. A total of 57 cohorts were investigated by the 39 studies (**Figure 2**). During analysis each different cohort was represented independently. Within these cohorts, age*–*specific prevalence estimates were represented individually if available. **Table 5** provides an overview of study characteristics by country. Population estimates are based on 2005 UNPD data [23].

**Table 5:** Study characteristics by country

Country	Proportion of regional adult (≥20) population (%)	No. of FPG & OGTT combined studies	No. of FPG*–*only studies	No. of OGTT*–*only studies	Total No. of studies
Afghanistan	1.28	0	0	0	0
Bangladesh	8.65	2	2	0	4
Bhutan	0.04	0	0	0	0
India	73.24	9	8	4	21
Iran	4.78	2	2	0	4
Maldives	0.02	0	0	0	0
Nepal	1.53	1	2	0	3
Pakistan	8.97	0	4	0	4
Sri Lanka	1.49	1	2	0	3
**Total**	**100.00**	**15**	**20**	**4**	39

FPG – fasting plasma glucose, OGTT – oral glucose tolerance test

The country with the largest number of studies, of all types, was by far India. It was also the only country for which suitable OGTT*–*only studies were available. No suitable studies of any kind were found in the literature search for three countries: Afghanistan, Bhutan, and the Maldives. The remaining five countries had a mixture of FPG*–*only and FPG+OGTT combined studies, with the exception of Pakistan, for which only FPG studies were found. The mean study size was 5178, with samples ranging from 331 to 25 969 participants. The median study year based on provided information was 2006 – while the earliest publication year was 1992, the earliest specified study year was 1998 and the most recent study year was 2009. More information about the study cohorts is given in **Table 6** and **Table 7**, and additional information is provided in **Online Supplementary Document(Online Supplementary Document)**.

**Table 6:** Cohort characteristics by country

Country	No. of FPG & OGTT combined cohorts	No. of FPG*–*only cohorts	No. of OGTT*–*only cohorts	Total No. of cohorts
Afghanistan	0	0	0	0
Bangladesh	2	3	0	5
Bhutan	0	0	0	0
India	20	9	6	35
Iran	2	2	0	4
Maldives	0	0	0	0
Nepal	1	2	0	3
Pakistan	0	4	0	4
Sri Lanka	1	5	0	6
**Total**	**26**	**25**	**6**	57

FPG – fasting plasma glucose, OGTT – oral glucose tolerance test

**Table 7:** General cohort characteristics

Characteristic	FPG & OGTT combined cohorts	FPG*–*only cohorts	OGTT*–*only cohorts	Total
Rural	9	13	3	25
Urban	15	7	3	25
Both/None	2	5	0	7
Minimum size	526	331	588	
Maximum size	12 514	25 969	1213	
Mean size	3580	4126	954	

FPG – fasting plasma glucose, OGTT – oral glucose tolerance test

## RESULTS

**Figure 3:**
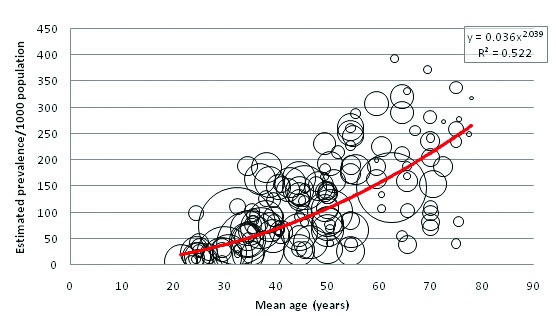
The relationship between crude prevalence of type 2 diabetes in Southern Asia and age.

**Figure 3** displays the relationship between mean age of sample and overall diabetes prevalence (both sexes combined, all diagnostic methods). A total of 151 individual data points for age*–*specific prevalence were plotted from 57 cohorts. 65 individual points were available from FPG and OGTT combined studies, 77 from FPG*–*only studies, and 9 from OGTT*–*only studies (**Tables 8****, ****9**** and ****10**). Age*–*specific prevalence data are provided in **Online Supplementary Document(Online Supplementary Document)**. **Figure 3** shows a positive association between mean age and diabetes prevalence. **Table 11** illustrates this association using the trend line equation derived from overall prevalence results and also displays population prevalence estimates based on 2005 UNPD estimates for the Southern Asia region. Based on these figures, the diabetes population prevalence for 2005 was estimated to be 7.47%. The 50*–*54 age group had the highest proportion of the burden at 11.31%, and the 20*–*24 group had the lowest proportion of the burden at 4.64%. A total of 51.3% of the burden was seen in those aged 50 or more. The diabetes burden increases with age until the ages of 50 and 54, after which it decreases. This is due to the population age structure in 2005 –diabetes prevalence per 1000 population is shown to increase continuously with age.

**Table 8:** Individual study prevalence data: FPG+OGTT combined studies

Authors	Country	Urban/rural	study sample size	Age range of participants	Mean age	Overall diabetes prevalence (/1000 population)	Male diabetes prevalence (/1000 men)	Mean age	Size of group	Female diabetes prevalence (/1000 women)	Mean age	Size of group
Bhowmik et al (2012) [24]	Bangladesh	Rural	2293	≥20	50	79	90	43.9	842	71	40.4	1451
Rahim et al (2008) [25]	Bangladesh	Rural	3954	≥20	37.1	70	75	39	1592	67	35.9	2375
Nazir et al (2012) [26]	India	Urban	2188	≥20	38.7	158	*–*	*–*	*–*	*–*	*–*	*–*
Prasad et al (2012) [27]	India	Urban	1178	20*–*80	45.6	157	178	47	590	138	44.2	588
Anjana et al (2011) [28]	India	Urban	1029	≥20	50*	137	*–*	*–*	*–*	*–*	*–*	*–*
		Rural	2480	≥20	50*	78	*–*	*–*	*–*	*–*	*–*	*–*
		Urban	1093	≥20	50*	109	*–*	*–*	*–*	*–*	*–*	*–*
		Rural	2476	≥20	50*	65	*–*	*–*	*–*	*–*	*–*	*–*
		Urban	840	≥20	50*	135	*–*	*–*	*–*	*–*	*–*	*–*
		Rural	2051	≥20	50*	30	*–*	*–*	*–*	*–*	*–*	*–*
		Urban	839	≥20	50*	142	*–*	*–*	*–*	*–*	*–*	*–*
		Rural	2247	≥20	50*	83	*–*	*–*	*–*	*–*	*–*	*–*
Deepa et al (2011) [29]	India	Urban	526	≥20	47.8	154	*–*	*–*	*–*	*–*	*–*	*–*
		Urban	596	≥20	41.1	153	*–*	*–*	*–*	*–*	*–*	*–*
Ravikumar et al (2011) [30]	India	Urban	2227	≥20	42.7	157	*–*	*–*	*–*	*–*	*–*	*–*
Ramachandran et al (2008) [31]	India	Urban	2192	≥20	38.2	186	209	38.2†	1053	167	38.2†	1139
		Urban	2290	≥20	36.8	164	171	36.8†	988	159	36.8†	1302
		Rural	2584	≥20	38	92	104	38.0†	1280	80	38.0†	1304
Zargar et al (2008) [32]	India	Rural	3024	20*–*40	30.8	25	*–*	*–*	*–*	*–*	*–*	*–*
Sadikot et al (2004) [33]	India	Urban	10 617	≥25	44.8	59	56	44.5	5379	58	45.1	5238
		Rural	7746	≥25	44.2	25	25	44.1	3629	25	44.3	4117
Ramachandran et al (2001) [34]	India	Urban	11 216	≥20	42.7	139	138	42.4	5288	140	43.0	5928
Hadaegh et al (2008) [35]	Iran	Urban	9489	≥20	43.5	142	141	44.8	4006	143	42.4	5483
Sadeghi et al (2007) [36]	Iran	Both	12 514	≥19	39	63	54	39†	6123	71	39†	6391
Shrestha et al (2006) [37]	Nepal	Urban	1012	≥40	54.7	191	246	54.9‡	423	151	54.5‡	589
Katulanda et al (2008) [38]	Sri Lanka	Both	4388	≥18	46.1	126	98	46.3	1720	109	46	2668

FPG – fasting plasma glucose, OGTT – oral glucose tolerance test

*Estimated mean age based on hypothetical maximum age of 80.

†Sex*–*specific mean ages not provided, overall mean age used for both sexes.

‡Estimated mean age based on age*–*group breakdowns.

**Table 9:** Individual study prevalence data: FPG*–*only studies

Authors	Country	Urban/Rural	Study sample size	age range of participants	mean age	Overall diabetes prevalence (/1000 population)	Male diabetes prevalence (/1000 men)	Mean age	Size of group	Female diabetes prevalence (/1000 women)	Mean age	Size of group
Rahman et al (2007) [39]	Bangladesh	Rural	975	≥20	38.9	85	94	41.7	360	80	37.3	615
Hussain et al (2005) [40]	Bangladesh	Rural	4757	≥20	37.5	23	19	39.7†	2030	25	35.8*	2720
		Urban	1555	≥20	33.5	81	77	35.9*	731	85	31.4*	824
Pandey et al (2013) [41]	India	Rural	2616	35*–*70	46.7	43	*–*	*–*	*–*	*–*	*–*	*–*
		Urban	2008	35*–*70	48.4	151	*–*	*–*	*–*	*–*	*–*	*–*
Vaz et al (2011) [42]	India	Rural	1266	≥20	39	103	84	39†	609	120	39†	657
Rao et al (2010) [43]	India	Rural	1239	≥30	51.3	160	188	50	434	144	52	805
Vijayakumar et al (2009) [44]	India	Rural	1645	≥18	47.2	146	165	48.2	624	135	46.2	1021
Namperumalsamy et al (2009) [45]	India	Both	25969	≥30	47	108	*–*	*–*	*–*	*–*	*–*	*–*
Chow et al (2006) [46]	India	Rural	4538	≥30	46.8	132	*–*	*–*	*–*	*–*	*–*	*–*
Gupta et al (2003) [47]	India	Both	1091	≥20	43.9	123	132	43.3	532	115	44.4	559
Misra et al (2001) [48]	India	Urban	532	≥18	35.4	103	112	37.8	170	99	34.3	362
Esteghamati et al (2009) [49]	Iran	Both	3397	25*–*64	44.2	87	*–*	*–*	*–*	*–*	*–*	*–*
Azimi*–*Nezhad et al (2008) [50]	Iran	Both	3438	20*–*64	48.5	55	*–*	*–*	*–*	*–*	*–*	*–*
Sharma et al (2011) [51]	Nepal	Both	14008	≥20	41.4	63	81	41.4†	5326	53	41.4†	8682
Paudyal et al (2008) [52]	Nepal	Rural	1475	≥40	54.7	41	*–*	*–*	*–*	*–*	*–*	*–*
Basit et al (2011) [53]	Pakistan	Rural	1264	≥25	42.3	142	165	43.5	424	131	41.7	840
Zafar et al (2011) [54]	Pakistan	Urban	1091	12*–*80	36	131	154	36†	293	123	36†	798
Mahar et al (2010) [55]	Pakistan	Urban	19211	30*–*90	42	87	*–*	*–*	*–*	*–*	*–*	*–*
Basit et al (2002) [56]	Pakistan	Rural	2032	≥25	38.9	72	119	40.4	670	49	38.1	1362
Pinidiyapathirage et al (2013) [57]	Sri Lanka	Urban	2986	35*–*64	52.3	247	233	52.2†	1349	259	52.3†	1637
Wijewardene et al (2005) [58]	Sri Lanka	Urban	4301	30*–*65	46.8	175	183	46.4	1891	168	47.2	2410
		Rural	571	30*–*65	44.6	175	73	44.7	275	67	44.6	297
		Rural	331	30*–*65	45.9	48	50	45.4	139	47	46.3	192
		Rural	844	30*–*65	45.7	71	70	45.6	387	72	45.8	457

FPG – fasting plasma glucose

*Estimated mean age based on age*–*group breakdowns.

†Sex*–*specific mean ages not provided, overall mean age used for both sexes.

**Table 10:** Individual study prevalence data: OGTT*–*only studies

Authors	Country	Urban/Rural	Study sample size	Age range of participants	Mean Age	Overall diabetes prevalence (/1000 population)	Male diabetes prevalence (/1000 men)	Mean age	Size of group	Female diabetes prevalence (/1000 women)	Mean age	Size of group
Boddula et al (2008) [59]	India	Urban	1112	≥30	55*	246	284	55*	557	207	55*	555
Ramachandran et al (2004) [60]	India	Rural	1213	≥20	41	63	74	41.8	497	56	40.5	716
Ramachandran et al (1994) [61]	India	Urban	873	≥60	70*	237	*–*	*–*	*–*	*–*	*–*	*–*
		Rural	588	≥60	70*	99	*–*	*–*	*–*	*–*	*–*	*–*
Ramachandran et al (1992) [62]	India	Urban	900	≥20	38	82	103	40	457	61	37	443
		Rural	1038	≥20	41	24	27	41	520	21	41	518

OGTT – oral glucose tolerance test

*Estimated mean age based on hypothetical maximum age of 80.

**Table 11:** Overall prevalence estimates for 2005

Age range	Mean age (years)	Prevalence / 1000 population (y = 0.0368x^2.039^)	2005 UNPD population estimates ( × 1000)	Calculated 2005 prevalence estimates ( × 1000)	Proportion of burden by age group (%)
20*–*24	22	20.09	152 031	3055	4.64
25*–*29	27	30.51	134 001	4088	6.21
30*–*34	32	43.14	115 491	4982	7.57
35*–*39	37	58.00	102 984	5973	9.07
40*–*44	42	75.10	89 614	6730	10.22
45*–*49	47	94.46	76 802	7255	11.02
50*–*54	52	116.09	64 131	7445	11.31
55*–*59	57	139.98	47 010	6581	9.98
60*–*64	62	166.16	37 303	6198	9.41
65*–*69	67	194.63	29 394	5721	8.69
70*–*74	72	225.40	20 538	4629	7.03
75*–*79	77	258.47	12 348	3191	4.85
		**Total:**	881 647	65 848	100.00
			2005 population prevalence:**7.47%**		

UNPD– United Nations Population Division

**Figure 4:**
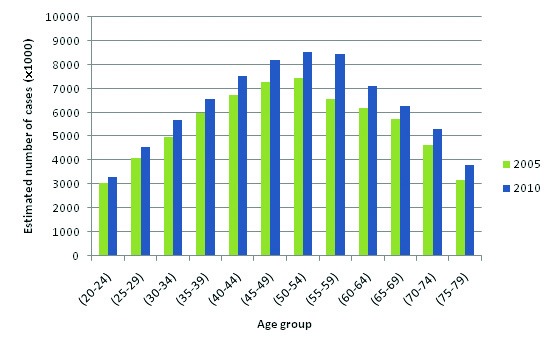
Estimated numbers of type 2 diabetes cases in Southern Asia (in thousands) by age group in 2005 and 2010.

**Figure 5:**
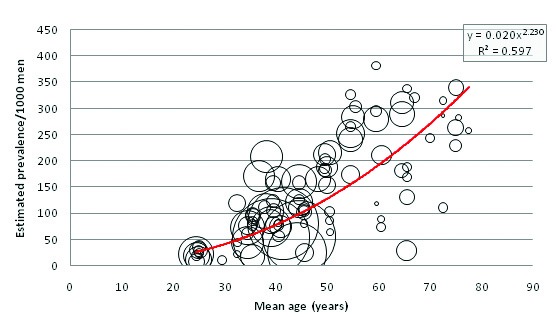
Relationship between crude prevalence of type 2 diabetes and age in Southern Asia in male examinees.

**Figure 6:**
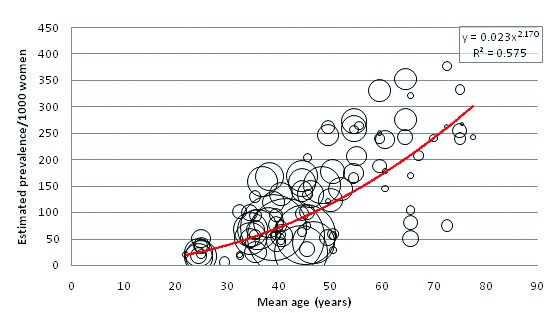
Relationship between crude prevalence of type 2 diabetes and age in Southern Asia in female examinees.

**Table 12** shows the overall prevalence equation applied to 2010 UNPD population estimates, and **Figure 4** compares the estimated numbers of diabetics between 2005 and 2010. A higher overall prevalence estimate is observed for 2010 than for 2005: 7.60% compared to 7.47%. **Figure 5** and **Table 13** illustrate the relationship between diabetes prevalence and age for males (all diagnostic methods). Based on the trend line equations, male prevalence is slightly higher than female prevalence at all ages. At the extreme, estimated male prevalence for the 70*–*79 age group is 336.08 per 1000, whereas female prevalence is 297.57 per 1000. **Figure 6** and **Table 14** illustrate female age*–*specific prevalence for all diagnostic methods. Sex*–* and age*–*specific prevalence data are presented in **Online Supplementary Document(Online Supplementary Document)**.

**Table 12:** Overall prevalence estimates for 2010

Age range	Mean age (years)	Prevalence / 1000 population (y = 0.0368x^2.039^)	2010 UNPD population estimates ( × 1000)	Calculated 2010 prevalence estimates ( × 1000)	Proportion of burden by age group (%)
20*–*24	22	20.09	163 363	3282	4.36
25*–*29	27	30.51	149 024	4546	6.04
30*–*34	32	43.14	131 390	5668	7.53
35*–*39	37	58.00	112 937	6550	8.70
40*–*44	42	75.10	100 399	7540	10.01
45*–*49	47	94.46	86 776	8197	10.89
50*–*54	52	116.09	73 608	8545	11.35
55*–*59	57	139.98	60 318	8444	11.21
60*–*64	62	166.16	42 750	7103	9.43
65*–*69	67	194.63	32 320	6291	8.35
70*–*74	72	225.40	23 627	5325	7.07
75*–*79	77	258.47	14 742	3810	5.06
		**Total:**	991 254	75 302	100.00
			2010 population prevalence: **7.60%**		

UNPD– United Nations Population Division

**Table 13:** Male prevalence estimates for 2005

Age range	Mean age (years)	Prevalence/1000 men(y = 0.0208x^2.2308^)	2005 UNPD male population estimates ( × 1000)	Calculated 2005 male prevalence estimates ( × 1000)	Proportion of burden by age group (%)
20*–*24	22	20.55	78 574	1614	4.07
25*–*29	27	32.45	69 157	2244	5.66
30*–*34	32	47.40	59 699	2830	7.14
35*–*39	37	65.53	53 302	3493	8.81
40*–*44	42	86.94	46 349	4029	10.17
45*–*49	47	111.73	39 741	4440	11.21
50*–*54	52	140.00	32 984	4618	11.65
55*–*59	57	171.82	23 669	4067	10.26
60*–*64	62	207.27	18 546	3844	9.70
65*–*69	67	246.42	14 386	3545	8.95
70*–*74	72	289.34	9985	2889	7.29
75*–*79	77	336.08	5979	2009	5.07
		**Total:**	452 371	39 622	100.00
			2005 male prevalence: 8.76%		
			2005 corrected prevalence: **7.72%**		

UNPD– United Nations Population Division

**Table 14:** Female prevalence estimates for 2005

Age range	Mean age (years)	Prevalence/1000 women (y = 0.0239x^2.1708^)	2005 UNPD female population estimates ( × 1000)	Calculated 2005 female prevalence estimates (1000)	Proportion of burden by age group
20*–*24	22	19.61	73 457	1441	4.10%
25*–*29	27	30.59	64 844	1984	5.65%
30*–*34	32	44.24	55 792	2468	7.03%
35*–*39	37	60.62	49 682	3012	8.58%
40*–*44	42	79.83	43 265	3454	9.84%
45*–*49	47	101.90	37 061	3777	10.76%
50*–*54	52	126.91	31 147	3953	11.26%
55*–*59	57	154.90	23 341	3616	10.30%
60*–*64	62	185.92	18 757	3487	9.93%
65*–*69	67	220.01	15 008	3302	9.41%
70*–*74	72	257.21	10 553	2714	7.73%
75*–*79	77	297.57	6368	1895	5.40%
		**Total:**	429 275	35 102	100.00%
			2005 female prevalence: 8.18%		
			2005 corrected prevalence: **7.20%**		

UNPD– United Nations Population Division

**Figure 7:**
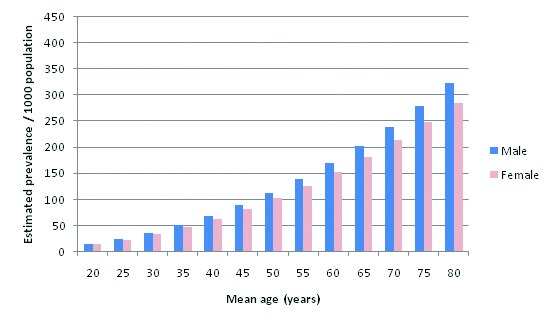
Male and female prevalence of type 2 diabetes in Southern Asia in 2005, adjusted to an “envelope” defined by all studies with prevalence data.

The crude population prevalence estimate for males in 2005 is 8.76% (39 622 000 cases), and for females is 8.18% (35 102 000 cases) (*P* < 0.001). These do not total the combined prevalence estimate of 65 848 000 cases (7.47%) since several studies did not provide enough data to calculate male and female age*–*specific prevalence, only enough to calculate combined sexes age*–*specific prevalence. To account for this incomplete data, the combined prevalence estimate was used as an envelope and a correction factor of 0.881 was applied to male and female prevalence estimates. Resultantly, the adjusted 2005 population prevalence estimates are 7.72% for males and 7.20% for females. **Figure 7** illustrates a comparison between corrected male and female prevalence estimates at 5*–*year age intervals.

### Urban and rural residency

**Figure 8:**
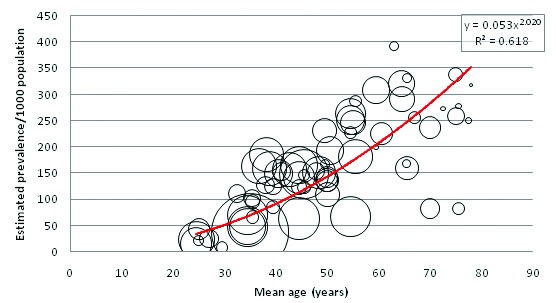
Crude prevalence of type 2 diabetes in Southern Asia in urban regions and its relationship with age.

**Figure 9:**
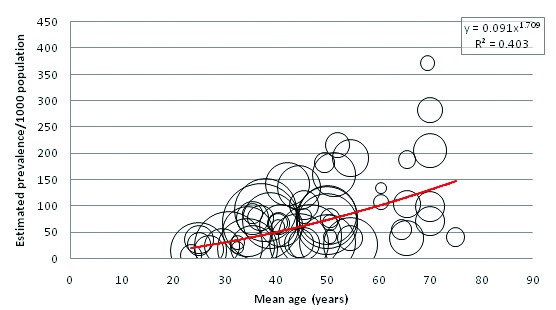
Crude prevalence of type 2 diabetes in Southern Asia in rural regions and its relationship with age.

**Figure 10:**
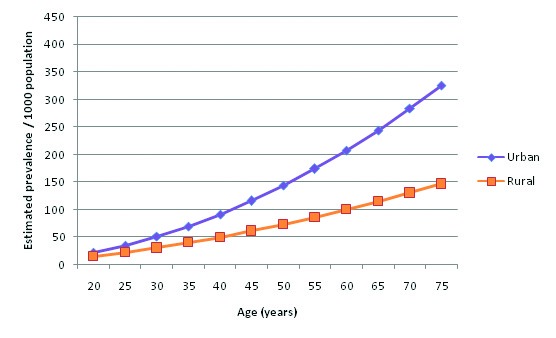
Comparison of the prevalence of type 2 diabetes in Southern Asia by age in urban and rural regions *–* both sexes included.

**Figure 11:**
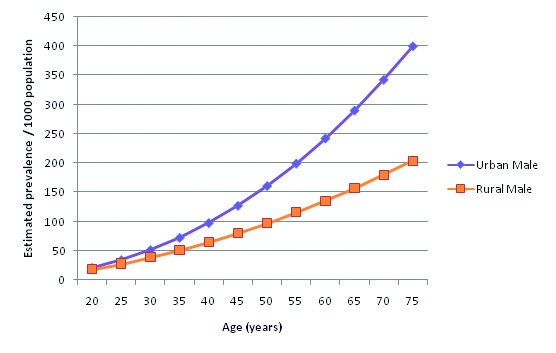
Comparison of the prevalence of type 2 diabetes in Southern Asia by age in urban and rural regions – men only.

**Figure 12:**
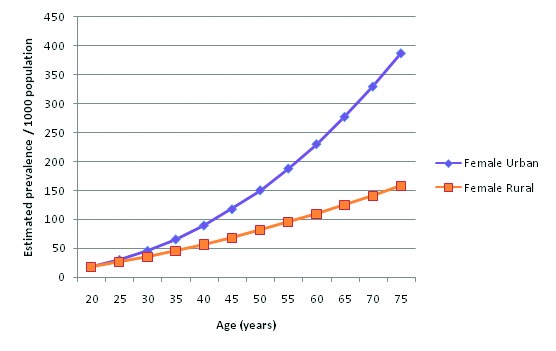
Comparison of the prevalence of type 2 diabetes in Southern Asia by age in urban and rural regions – women only.

**Figure 13:**
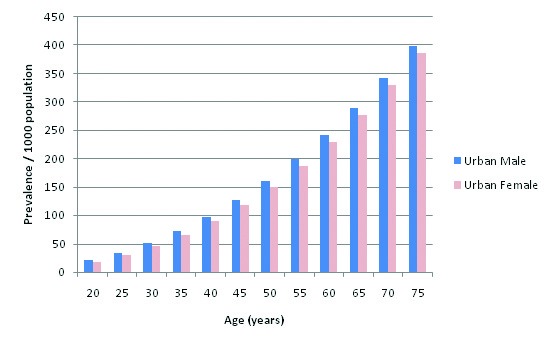
Male and female prevalence of type 2 diabetes in Southern Asia based on information from urban regions.

**Figure 14:**
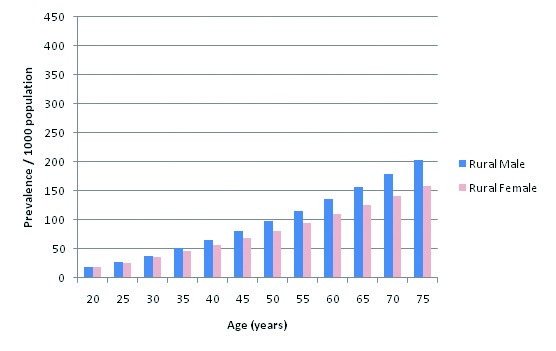
Male and female prevalence of type 2 diabetes in Southern Asia based on information from rural regions.

32 out of the 39 selected studies (50 out of 57 cohorts) specifically defined their study population as residing in an urban area or a rural area. Incidentally, out of the reporting studies, 25 cohorts were specified as urban and 25 as rural. **Figure 8** illustrates diabetes prevalence against age in urban cohorts (both sexes, all diagnostic methods), and **Figure 9** does likewise for rural cohorts. **Table 15** and **Figure 10** highlight the prevalence differences observed between urban and rural cohorts. **Figure 11** compares prevalence of diabetes in urban males with prevalence in rural males. **Figure 12** compares prevalence of urban and rural females. In both cases urban residency is associated with significantly higher diabetes prevalence (*P* < 0.001). **Figures 13**** and ****14** show the estimates for both sexes by residency. Individual bubble graphs for residency are presented in **Online Supplementary Document(Online Supplementary Document)**.

**Table 15:** Urban and rural overall prevalence comparison (*P* < 0.001)

Age (years)	Estimated urban prevalence/1000 population (y = 0.053x^2.0206^)	Estimated rural prevalence/1000 population (y = 0.0917x^1.7091^)
20	22.55	15.34
25	35.40	22.47
30	51.16	30.68
35	69.86	39.93
40	91.50	50.17
45	116.08	61.36
50	143.62	73.46
55	174.12	86.46
60	207.59	100.32
65	244.03	115.03
70	283.45	130.56
75	325.86	146.90

### Method of diabetes diagnosis

**Figure 15:**
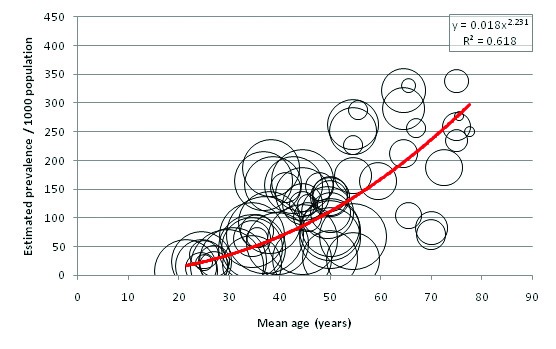
Relationship between crude prevalence of type 2 diabetes and age in Southern Asia based on the studies using FPG+OGTT in their case definition. FPG – fasting plasma glucose, OGTT – oral glucose tolerance test.

**Figure 16:**
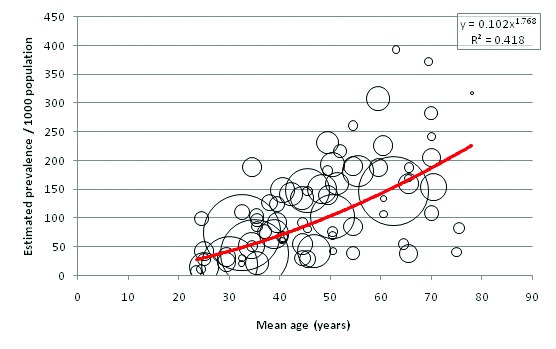
Relationship between crude prevalence of type 2 diabetes and age in Southern Asia based on the studies using FPG only in their case definition. FPG – fasting plasma glucose.

**Figure 17:**
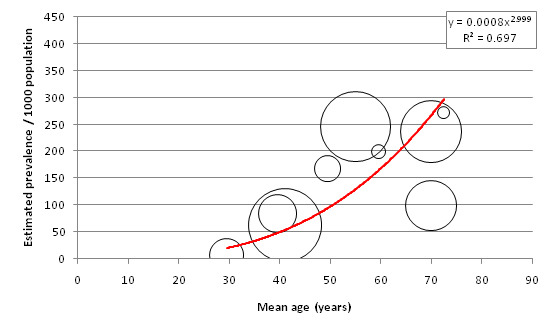
Relationship between crude prevalence of type 2 diabetes and age in Southern Asia based on the studies using OGTT only in their case definition. OGTT – oral glucose tolerance test.

**Figure 18:**
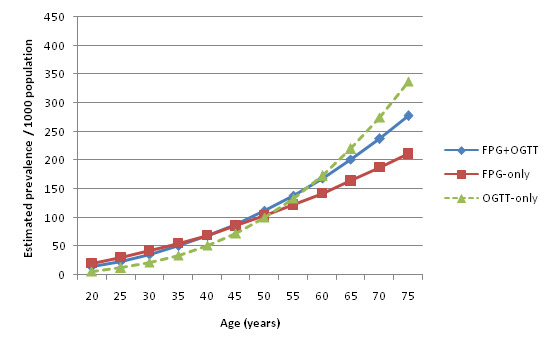
Comparison of the relationship between crude prevalence of type 2 diabetes and age in Southern Asia depending on the diagnostic methods used to establish case definition.

**Figure 15** shows the relationship between age and diabetes prevalence when only considering studies that utilised both FPG and OGTT in diagnosing diabetes. **Figure 16** shows the relationship for studies that used only FPG, and **Figure 17** shows OGTT*–*only studies. 65 age*–*specific data points were used to calculate the FPG+OGTT trend line, 77 data points used to calculate the FPG*–*only trend line, but due to the small number of available OGTT studies only 9 age*–*specific data points were used in calculating the OGTT trend line. **Figure 18** compares the estimated prevalence using each of the three diagnostic methods. The combined FPG plus OGTT studies resulted in a higher population prevalence estimate than FPG*–*only studies when applied to UNPD figures, as shown by **Table 16** and **Table17**. FPG plus OGTT studies result in a population prevalence of 7.75%, while FPG*–*only studies result in a prevalence of 7.32%. The small number of OGTT*–*only studies predicted a population prevalence of 6.95%, as shown in **Table 18**.

**Table 16:** Prevalence estimates for 2005 based on FPG+OGTT studies

Age range	Mean age (years)	Prevalence/1000 population (y = 0.0181x^2.2319^)	2005 UNPD population estimates ( × 1000)	Calculated 2005 prevalence estimates ( × 1000)	Proportion of burden by age group (%)
20*–*24	22	17.94	152 031	2727	3.99
25*–*29	27	28.34	134 001	3797	5.56
30*–*34	32	41.40	115 491	4782	7.00
35*–*39	37	57.25	102 984	5895	8.63
40*–*44	42	75.96	89 614	6807	9.96
45*–*49	47	97.64	76 802	7499	10.97
50*–*54	52	122.36	64 131	7847	11.48
55*–*59	57	150.18	47 010	7060	10.33
60*–*64	62	181.18	37 303	6759	9.89
65*–*69	67	215.42	29 394	6332	9.27
70*–*74	72	252.96	20 538	5195	7.60
75*–*79	77	293.86	12 348	3629	5.31
		**Total:**	881 647	68 330	100.00
			FPG+OGTT population prevalence: **7.75%**		
			Overall population prevalence (2005): 7.47%		

FPG – fasting plasma glucose, OGTT – oral glucose tolerance test, UNPD– United Nations Population Division

**Table 17:** Prevalence estimates for 2005 based on FPG*–*only studies

Age range	Mean age (years)	Prevalence/1000 population (y = 0.1021x^1.7684^)	2005 UNPD population estimates ( × 1000)	Calculated 2005 prevalence estimates ( × 1000)	Proportion of burden by age group
20*–*24	22	24.15	152 031	3672	5.69%
25*–*29	27	34.69	134 001	4649	7.20%
30*–*34	32	46.85	115 491	5411	8.38%
35*–*39	37	60.57	102 984	6237	9.66%
40*–*44	42	75.78	89 614	6791	10.52%
45*–*49	47	92.46	76 802	7101	11.00%
50*–*54	52	110.56	64 131	7090	10.98%
55*–*59	57	130.05	47 010	6114	9.47%
60*–*64	62	150.90	37 303	5629	8.72%
65*–*69	67	173.08	29 394	5088	7.88%
70*–*74	72	196.58	20 538	4037	6.25%
75*–*79	77	221.36	12 348	2733	4.23%
		**Total:**	88 647	64 553	100.00%
			FPG*–*only population prevalence: **7.32%**		
			Overall population prevalence (2005): 7.47%		

FPG – fasting plasma glucose, UNPD– United Nations Population Division

**Table 18:** Prevalence estimates for 2005 based on OGTT*–*only studies

Age range	Mean age (years)	Prevalence/1000 population (y = 0.033x^2.1118^)	2005 UNPD population estimates ( × 1000)	Calculated 2005 prevalence estimates ( × 1000)	Proportion of burden by age group
20*–*24	22	8.52	152 031	1295	2.11
25*–*29	27	15.74	134 001	2109	3.44
30*–*34	32	26.21	115 491	3026	4.94
35*–*39	37	40.51	102 984	4172	6.81
40*–*44	42	59.25	89 614	5309	8.66
45*–*49	47	83.03	76 802	6377	10.41
50*–*54	52	112.44	64 131	7211	11.77
55*–*59	57	148.09	47 010	6962	11.36
60*–*64	62	190.58	37 303	7109	11.60
65*–*69	67	240.51	29 394	7070	11.54
70*–*74	72	298.47	20 538	6130	10.00
75*–*79	77	365.07	12 348	4508	7.36
		**Total:**	881, 647	61 278	100.00
			OGTT*–*only population prevalence: **6.95%**		
			Overall population prevalence (2005): 7.47%		

OGTT – oral glucose tolerance test, UNPD– United Nations Population Division

## DISCUSSION

This study provides the most up*–*to*–*date transparent estimation of diabetes prevalence in the UN Southern Asia region, building upon previous studies looking at prevalence in comparable regions or in specific constituent countries [21,22,63,64]. It is also, to our knowledge, the first study to transparently estimate diabetes prevalence and trends in Southern Asia by synthesizing findings from numerous community*–*based studies in addition to broader national population studies.

A transparent systematic literature review of two online databases was carried out. Pre*–*defined inclusion and quality criteria narrowed down 39 studies from an initial 5653 results. Search terms were specified, and quality assessment criteria for selected studies were provided in Appendices. Enough studies were captured for estimation of overall diabetes prevalence, male and female prevalence, and urban and rural prevalence for each sex. Study cohort sites had a wide geographic distribution within the countries that were analysed, as shown in **Figure 2**. Additionally there was an even split of rural and urban studies, allowing for comparison of prevalence in both demographics. Data was not captured from all countries in the Southern Asia region –the systematic literature review did not find any suitable studies from Afghanistan, Bhutan, or the Maldives. However, **Table 5** shows that these three countries have the smallest adult populations in the region. For all other countries, including the three most populous countries of India, Pakistan, and Bangladesh, suitable numbers of geographically dispersed studies were identified. While this paper was able to adequately meet most of its set objectives, the small number of OGTT*–*only studies captured in the search meant that all recognised diagnostic methods could not be fully compared in terms of prevalence estimates, and so this objective was only partially met.

By applying this study’s prevalence estimates to UNPD population figures [23], the overall diabetes prevalence for the Southern Asia region was estimated to be 7.47% for 2005, and 7.60% for 2010. Although the reviewed studies were more representative of the year 2005, it is interesting to note the effects of an ageing population on diabetes prevalence. Estimates indicate that 25.0% of the regional population was aged 50 or older in 2010, compared to 23.9% in 2005. In addition, UNPD projections for Southern Asia predict that total population and life expectancy at birth for both males and females will continue to rise in the region over the next 30 years [23]. The findings of this study suggest that as the population continues to age in the future, the overall burden of diabetes in Southern Asia will also continue to increase, and that concerted policy action is needed to facilitate the response to this increased burden.

It was found that diabetes prevalence was consistently higher for males than for females. The burden was highest in the 50*–*54 age group for both sexes, and within this age group there was a 9.35% difference between estimated male and female prevalence. Prevalence/1000 population continued to increase with age for both sexes but due to the population age structure the burden attributed to older age groups was progressively smaller after the ages of 50*–*54. After correction for missing data, the 2005 population prevalence estimate for males was 7.72%, and for females was 7.20%. This translates into an estimated 34 915 000 male and 30 933 000 female diabetics in Southern Asia in 2005.

This study found that urban residency was strongly associated with higher diabetes prevalence for both sexes. The observed difference is noteworthy – past the age of 55 the urban prevalence was estimated to be more than twice the rural prevalence. Although males had higher prevalence than females in both urban and rural settings, the difference was noticeably smaller in urban cohorts than rural cohorts: a 6.03% difference between urban males and females, but a 16.74% difference between their rural counterparts. Higher rates of diabetes among urban residents may be explained through increases in physical inactivity and consumption of high sugar and fat diets – both strong risk factors for diabetes – that have become synonymous with urban lifestyles. Mohan suggested that diabetes rates in India are quickly escalating because of the rapid urbanisation that is sweeping the country [17]. Conversely, rural prevalence remains lower because of limited exposure to these risk factors and maintenance of traditional physically vigorous rural lifestyles.

Sufficient numbers of FPG*–*only and FPG+OGTT combined studies were identified to allow comparison between these two diagnostic methods. The small number of OGTT*–*only studies also provided interesting trends. Overall the differences between these methods appeared to be minimal. The higher prevalence estimates of FPG+OGTT compared to FPG*–*only were to be expected since the former used an additional diagnostic method. FPG*–*only studies estimated higher diabetes prevalence at younger ages (<52) and lower prevalence at older ages (>52) compared to OGTT*–*only. Combined FPG+OGTT prevalence estimates lay in between the FPG and OGTT estimates at both ends of the age spectrum. In addition, FPG*–*only studies estimated the highest burden proportion to be at a younger age – in the 45*–*49 age group – followed by a small decrease in the 50*–*54 age group. FPG+OGTT studies and the limited number of OGTT*–*only studies found highest burden proportion in the 50*–*54 age group. These findings suggest that FPG may have greater sensitivity at younger ages, while OGTT may be more sensitive to diabetes in older age.

This study’s prevalence estimates were primarily based on trend line equations obtained by plotting study size, mean age and prevalence estimates on bubble graphs. Bubble graphs accommodate gaps in data better than weighted mean box*–*plots, and the resultant trend lines can be used to estimate the expected prevalence for any given age rather than just the specific age group means. Therefore, bubble graphs are preferable over weighted mean box*–*plots when considering a disease such as diabetes, for which the steady prevalence increase with age has previously been well established [21]. The trend lines obtained in this study all had high R^2^ values – specified on each graph for purposes of transparency – indicating that they were representative of the data and took into account a high degree of variance.

Several of this study’s findings are in line with previous estimations of diabetes burden in comparable regions. Estimates for 2000 [21] and 2010 [22] both suggest that in developing countries, diabetes burden is highest between the ages of 40 and 64, and lowest under the age of 40. This was reflected in the findings. Wild and colleagues also found that global diabetes prevalence was higher overall for males than females. However, more recent estimates for Southern Asia found no distinct increase in diabetes risk with male gender [64]. This study found a small but consistent difference between male and female prevalence. Based on national surveys for countries in the region, Jaywardena and colleagues estimated the overall Southern Asia diabetes prevalence to be in the range of 4.5*–*10.3% for the period 1995*–*2006[64]. This study’s estimate of 7.47% for 2005 falls in the middle of this range. Additionally, this study’s 2010 prevalence estimate of 7.60% is similar to the findings of Shaw and colleagues for the WHO region of South*–*East Asia[22], a geographic region that includes six out of the nine countries of the UN Southern Asia region (all apart from Afghanistan, Iran and Pakistan). Recent IDF estimations suggest that diabetes prevalence may be even higher – at an estimated 8.60% for South*–*East Asia in 2011 [65]. However, it is difficult to make comparisons between estimates for the UN Southern Asia region and the WHO South*–*East Asia region. In addition, different studies often use widely varying methods for study selection and estimating prevalence, contributing to the observed inconsistencies. Nevertheless, a substantial body of evidence including this study indicate that the diabetes burden in this area of the world is large and growing.

### Limitations

This systematic review considered published studies from 1980*–*2013. However, suitable studies were only identified for the period 1992*–*2013. Not looking at studies prior to 1980 may have excluded viable studies, but older studies often used previous diabetes diagnostic criteria which underestimated prevalence. Most of the identified studies carried out prior to 1990 used the old 1980/1985 WHO criteria with the higher FPG cut*–*off of 7.8mmol/L for diabetes diagnosis. Including such studies in the analysis would distort the prevalence estimate because of the differing diabetes case definitions.

Several recent studies did not detail the diagnostic criteria used to identify diabetics, despite explaining their method of diagnosis. These studies were excluded to ensure that the case definition of diabetes in the selected studies remained constant. Other studies did not make it clear whether the biochemical samples they used were venous or capillary, or whether whole blood or plasma was analysed. WHO provides diagnostic guidelines for each of these sample types [11,12], but when sample type was not specified the study was excluded to minimise case definition misclassifications. Adherence to these stringent quality assessment criteria potentially limited the number of studies that could be included in this analysis. In addition, studies that did not diagnose diabetes through biochemical measurements but instead used techniques such as self*–*reported surveys were also excluded. Several studies found low knowledge of diabetes in Southern Asia, even in diabetic patients [66,67], and therefore such methods were considered unreliable.

Studies investigating other forms of diabetes such as gestational diabetes or diabetes insipidus were excluded. These studies were easy to identify because of the specific nature of gestational diabetes, and the different clinical presentation of diabetes insipidus to diabetes mellitus (DM). However, a major limitation of this paper was the inadequate ability to distinguish between type 1 DM and type 2 DM. Many risk factors for type 2 DM, such as diet and physical inactivity, are modifiable and therefore may be amenable to policy intervention, but there are no known preventative measures against type 1 DM. Most analysed studies did not further investigate identified diabetics to exclude type 1 DM, meaning that this study’s findings may be an overestimation of the prevalence of type 2 DM. However, in the adult age range that was being investigated, type 2 DM is more common than type 1 DM, hence their previous names of “adult*–*onset diabetes mellitus” and “childhood*–*onset diabetes mellitus”. In addition, it has been noted that while type 1 DM rarely causes death by ketoacidosis in developed countries, sufferers in many developing countries may unfortunately have a radically shortened lifespan due to limited insulin availability which is crucial for type 1 DM management [68]. This may hold true especially for some of the poor rural areas investigated in this study. As a result, any error in the prevalence estimate due to type 1 diabetics is likely to be small.

Only published studies were reviewed. The resulting analysis may have suffered from publication bias as the reviewed papers may only show those studies in which significant results were found. Publication bias may have prevented studies that did not show significant diabetes prevalence from being published in the first place, preventing these studies from being captured in this review. While no limits were set on language, time constraints also prevented translation and therefore inclusion of non*–*English studies. This may have resulted in exclusion of viable non*–*English studies. However, even without setting language limits, only a very small number of non*–*English studies were identified by the literature search. This might be due to the status of English as an official language in several ex*–*colonial countries in the Southern Asia region, most notably India and Pakistan.

No suitable studies were identified for three out of nine of the countries in the region – Afghanistan, Bhutan, and the Maldives. As mentioned, however, these are the least populous countries in the region, and combined only account for 1.34% of the regional adult population. Nevertheless it is a noteworthy limitation that no data was available for these countries when the estimated regional population prevalence took their populations into account as well. Another limitation arose when comparing urban and rural studies. Cohorts were classified as urban or rural on the basis of individual study descriptions. No standardised definitions of ‘urban’ or ‘rural’ were used, meaning there may be discrepancies between different studies on their cohort classification.

Not all selected studies provided male and female sex*–*specific prevalence breakdowns, while others did not provide sample sizes for sex*–*specific prevalence or did not report mean age. This limited the number of cohorts that could be analysed for male and female age*–*specific prevalence. Where possible, sample sizes were calculated based on reported number of cases and corresponding prevalence figures. However, this study’s findings were limited by assumptions that had to be made to account for incomplete data. UNPD national age structures were applied where appropriate [23]. This method of estimation may have increased imprecision as study populations are not necessarily representative of the national average. The use of a correction factor to account for incomplete sex*–*specific data may have been another source of imprecision. A hypothetical maximum age of 80 was used to calculate mean age for studies that provided no maximum age range. This assumption was made as this was the highest age used in reviewed studies that included a maximum age, and also because the minority of studies that looked at participants aged 80+ had very small sample sizes for those age groups. In addition, the 2005 UNPD population estimates for Southern Asia suggest that the 80+ age group accounts for only 0.58% of the population, so this was not considered a major limitation.

While every effort was made to ensure accuracy and the use of systematic methods, human error may have resulted in accidental exclusion of relevant studies when inclusion and quality criteria were being applied. Having only one person review and evaluate studies is a potential limitation of this study design. Using several independent evaluators to select studies, with a suitable method for resolving disputes, may increase reliability of the study’s findings.

### Implications for policy

Evidence on disease burden is essential for countries to plan and develop programs in response to the NCD pandemic. The WHO 2008*–*2013 Action Plan for the Prevention and Control of Noncommunicable Diseases highlighted that before prevention and control policies can be implemented, one of the first steps is to assess the burden of disease [69]. Engelgau and colleagues also proposed a framework for policy decision*–*making on NCDs [7], and improved surveillance is an essential component of their initial assessment stage. Several Southern Asian countries have shown improvements in their national NCD surveillance and monitoring capabilities in the last decade [2]. However, as previously discussed, estimates of diabetes burden vary widely between different sources. Further improvements in national surveillance capabilities are needed so that authoritative and standardised estimations of the burden of NCDs can be made. Accurate and up*–*to*–*date estimations of burden are also important in evaluation of current policies, programs, and of health system capacity. A number of policies have shown promise – in 2003 India enacted The Cigarette and Other Tobacco Products Act which is considered to have effectively reduced the public’s exposure to tobacco smoke, through advertisement, and minors’ access to cigarettes [7]. India also recently launched a pilot phase of the National Programme for Prevention and Control of Diabetes, Cardiovascular Diseases and Stroke (NPDCS), and has made financing commitments for the near future [70]. The aims of this program are laudable but monitoring of such programmes is necessary to ensure resources are used efficiently, and that appropriate targets and priorities are set [7]. This is of special importance due to rapidly*–*changing nature of NCDs and the many challenges that governments of developing Southern Asian countries face when attempting to deal with them.

The findings of this study suggest that future urbanisation and increased life expectancy will lead to a substantial rise in the burden of type 2 DM in the Southern Asia region. Commentators have noted that the process of population ageing currently being observed in developing countries is different to the demographic transition that occurred in developed countries several decades ago. In particular, the current demographic transition in developing countries has occurred on a faster scale than in developed countries, and without the associated improvements in living conditions, social provisions, and access to health care [7]. This has led to a ‘compressed timeline’ for developing countries to mount effective responses to growing NCD burdens compared to what developed countries had [1]. ‘Unhealthy ageing’ in Southern Asia due to these lagging improvements in nutrition and socioeconomic conditions is predicted to add to the natural increases in disease burden expected of an ageing population [7]. Therefore it is paramount that both prevention and treatment policy options are considered – the root causes of NCDs need to be addressed, and health system capacity should be reviewed to deal with the increasing burden.

Prevention efforts for diabetes can come in many forms, but there are several that may be especially relevant to Southern Asia. Before any successful prevention policies can be implemented it is important that knowledge and awareness regarding diabetes is improved in the general population. Many studies have shown that in Southern Asian populations, awareness of diabetes and its risk factors is poor [71,72], even among diabetic patients [66,67]. Population*–*level education and health promotion schemes should be put into place to improve awareness of the risk factors for diabetes and the other main NCDs. Diabetes has many lifestyle*–*modifiable risk factors, and by improving knowledge regarding these, the Southern Asian population can be empowered to pursue healthier lifestyle choices. Increased risk factor awareness and the resulting community empowerment have been seen to have a positive effect in the past. Mohan and colleagues reported that following such efforts, an Indian community was prompted to create a public park with their own funds which resulted in significant improvements in physical activity levels for local resident [73].

Departure from traditional dietary patterns and the uptake of diets high in saturated fats and heavily refined carbohydrates are believed to be important underlying factors in rising rates of obesity and diabetes in Southern Asia [17]. In particular, low intake of fibre, mono*–* and poly*–*unsaturated fats, and high consumption of refined carbohydrates, saturated fats and trans*–*fats have been identified as problematic dietary habits leading to insulin resistance in Southern Asian populations [74]. Policies should focus on addressing these unhealthy dietary patterns with a view to inform and educate. Successful policies from the health sector may include efforts to improve food labelling through dialogue with food companies, which when combined with education on NCD dietary risk factors may go some way to lowering diabetes and obesity incidence [7]. Focus should also be given on encouraging people to switch from traditional high trans*–*fat cooking oils such as *ghee* and *vanaspati* to poly*–*unsaturated alternatives [74].

The health sector has an important role to play in the management and treatment of NCDs. **Table 2** shows that while all the Southern Asian economies are growing, many of them spend very small proportions of their gross domestic product (GDP) as health expenditure. In 2001, the WHO Commission on Macroeconomics and Health found that a basic set of essential health interventions costs approximately US$34 per capita, believed to be a modest sum even for low*–*income countries [75]. However, several Southern Asian countries spend less on health than even this recommended minimum per capita expenditure [20]. NCDs undermine economic progress and have significant macroeconomic and microeconomic impacts [76]. The increasing burden of NCDs will strain existing health systems if health expenditure is not increased. Physician density is also low in many Southern Asian countries; Engelgau and colleagues suggest that improving region*–*wide health education and training capacities may help fill human resource gaps across the region by taking advantage of economies of scale [7].

Even if health system capacities are expanded to deal with the increasing burden of NCDs, access to appropriate health care remains a major challenge across the Southern Asia region. Studies in several Southern Asian countries have found significant personal expenditure and substantial financial loss associated with paying for diabetes treatment, with the main costs being the direct expenses of investigation, treatment, and hospital admission [49,77,78]. These expensive out*–*of*–*pocket medical costs are a major barrier for universal access to health care services in Southern Asia, and result in widening inequalities between rich and poor. In addition to increasing health care capacity, Southern Asian countries should aim to improve access to health care by implementing WHO universal coverage reforms [79]. Successful policy strategies may involve improving revenue collection by targeting tax avoidance; pooling risk to reduce cost*–*sharing; efficiency savings from introducing Health Technologies Assessment (HTA); and simply increasing the priority given to health and thereby increasing governmental health expenditure.

### Implications for future research

Following the recent WHO addendum approving the use of HbA1_c_ as a diagnostic method [1], WHO and American Diabetes Association diagnostic guidelines and criteria are mostly aligned. Future studies investigating prevalence in a population or community should use these standardised methods and criteria for diagnosing diabetes to allow for informative comparison between studies. The utilisation of HbA1_c_ measurements also presents new avenues for diabetes epidemiological research. If appropriate quality assurance measures are put in place, as per WHO recommendations, HbA1_c_ presents a valuable method for investigating long term changes in glycaemic status in study subjects.

Studies calculating overall prevalence inadvertently calculate male and female prevalence as well, but as seen in this paper, in many cases these were not reported. Additionally many studies failed to report basic information such as mean age of sample, age group of sample, type of biochemical sample taken, or diagnostic criteria used to define diabetes. Access to this information would reduce the number of assumptions that have to be made for incomplete data, and additionally would allow for a more accurate estimate as far fewer studies would have to be excluded from analysis. As awareness of the need for large*–*scale population estimates of NCDs becomes more commonplace, it is hoped authors carrying out community*–*based studies begin to employ common standards to allow effective utilisation of their work in burden of disease analyses.

Diabetes is one end of a spectrum of glycaemic states, and future studies could estimate the burden of different forms of prediabetes as well. Impaired fasting glucose (IFG) and impaired glucose tolerance (IGT) are diagnosed with FPG and OGTT respectively so many studies report their prevalence alongside diabetes. Regional estimation of the burden of both prediabetes as well as diabetes would allow for a more comprehensive analysis of the challenges these hyperglycaemic diseases pose.

This study found that diabetes is strongly associated with urban residency in Southern Asia. As the region continues a process of rapid globalisation and urbanisation, it is important to maintain diabetes surveillance in both urban and rural cohorts. Urban migration and the increasing accessibility of inactive, sedentary lifestyles suggest that the diabetes burden will increase in the future. Projections for 2030 predict that several Southern Asian nations will continue to rank among the countries with the largest numbers of diabetic residents [21,22]. However, monitoring and analysis of these vulnerable populations can inform public health policymakers and help manage the burden of diabetes. It is important that future studies focus on high*–*risk populations: urban residents, and, as life expectancy increases, the growing number of elderly people as well.

## CONCLUSION

This systematic literature review found a high prevalence of type 2 DM in the Southern Asia region. Diabetes prevalence was associated with male gender, and strongly associated with older age and urban residency. On the basis of these findings this study also predicted that diabetes prevalence will continue to increase in the future as life expectancy in the region rises and countries continue to undergo processes of urbanisation. The findings of this study were consistent with several past studies, but dissimilar to the results of others. This highlights that inconsistent surveillance and conflicting estimations of burden are some of the many challenges faced by the Southern Asia region and its constituent countries in their effort to respond to the rising burden of NCDs.

Accurate and up*–*to*–*date estimates of burden of disease are essential for planning of policies, target and priority setting, as well as monitoring and assessment of existing programs. However, greater standardisation and shared principles are needed across different studies so that strong, clear messages are given to policymakers. It is hoped that improved surveillance capabilities in Southern Asian countries will encourage common standards for prevalence estimation to be established.

While current policies and programs on diabetes control have met with some success, the region faces numerous hurdles. Rising life expectancies coupled with ‘unhealthy’ ageing present a new set of challenges to those faced by developed countries several decades ago. Policies focusing on prevention have to deal with a population that largely has little awareness of diabetes and its risk factors, and is becoming increasingly accustomed to a sedentary lifestyle and unhealthy eating patterns. Health sectors also have their own set of issues – total health expenditure is low in many Southern Asian countries, there are significant human resource gaps, and already struggling health systems are predicted to be put under even greater strain as diabetes prevalence continues to increase. In addition, from an equity perspective, high cost*–*sharing coupled with the long*–*term care needed for NCDs such as diabetes means that access to health care may be limited for a large proportion of people in the region.

However, despite these numerous policy challenges and the projected increase in diabetes prevalence, slow progress is being made. NCDs are at the forefront of the international health agenda, several Southern Asian countries have greatly improved their NCD surveillance and monitoring capacities, and the numbers of studies estimating burden of type 2 diabetes mellitus appears to be increasing in recent years. Greater attention needs to be paid to this disease and its risk factors on national and regional levels in Southern Asia so that the growing burden of diabetes can be adequately addressed in the future.
