# Supplementary material for: Urbanization and prevalence of type 2 diabetes in Southern Asia: A systematic analysis
Source: J Glob Health. 2014 Jun;4(1):010404. doi: 10.7189/jogh.04.010404 (PMC4073245; doi:10.7189/jogh.04.010404)
Supplement: Online Supplementary Document [file jogh-04-010404-s001.pdf]

Online Supplementary Document

Cheema A et al. Urbanization and prevalence of type 2 diabetes in Southern Asia: A systematic analysis
Journal of Global Health 2014;4:010404

I. Notes on Diabetes Diagnostic Methods

II. Notes on Data Analysis

III. Quality Assessment – FPG & OGTT combined studies

IV. Quality Assessment – FPG only studies

V. Quality Assessment – OGTT only studies

VI. Age-Specific Prevalence Data – Both Sexes Combined

VII. Age-Specific Prevalence Data – Males

VIII. Age-Specific Prevalence Data – Females

IX. Sex-specific Bubble Graphs for Residency

I. Notes on Diabetes Diagnostic Methods

As mentioned in the main text, the two main diagnostic methods used for diagnosing diabetes are the Fasting Plasma Glucose (FPG) test and the Oral Glucose Tolerance Test (OGTT). In clinical settings the gold standard method for measuring FPG involves taking a venous plasma sample and laboratory testing for glucose. However, due to the associated storage and measurement difficulties, epidemiological studies often instead measure capillary values using glucometers [12].

The OGTT is carried out on fasting patients and involves measurement of baseline blood glucose, followed by ingestion of 75g anhydrous glucose, and a subsequent blood glucose measurement after two hours to determine the efficacy with which glucose has been eliminated from the patient's blood [12].Generally OGTT is more difficult to carry out than FPG in epidemiological studies, particularly those with large samples, as the subjects are required to wait for two hours for the second blood glucose measurement. For individual cases in a clinical setting, two abnormal measurements from either test are required for a positive diabetes diagnosis, but again due to the limitations of epidemiological studies, only one abnormal measurement may be used [12].

The American Diabetes Association (ADA) discourages use of OGTT for diagnostic purposes due to its relative inconvenience, greater cost, and lower reproducibility [10]. However, WHO continues to recommend its use as a diagnostic test as some studies suggest OGTT diagnoses a different subset of people than FPG, and others indicate that people diagnosed on the basis of OGTT may have higher morbidity and worse prognostic outcomes [11]. WHO has also recently advocated the measurement of glycated haemoglobin ($HbA1_C$) for diagnostic purposes, which was previously ruled out due to measurement accuracy problems [13]. As this is only a recent addendum, and due to the stringent quality assurance tests necessitated when measuring $HbA1_c$ under these new guidelines, its use in epidemiological studies has been limited to date.

II. Notes on Data Analysis

For purposes of modelling and analysis, data on mean age, sample size, and age-specific prevalence was required. Some studies did not provide age-specific prevalence estimates, age-group sample sizes, or mean ages for their samples. Wherever possible these were calculated from the available data. For studies that provided the number of diabetes cases in an age group and the corresponding prevalence percentage, sample sizes were simply calculated by dividing 100 by the prevalence percentage and multiplying the result with the number of cases. However, some studies only gave age-specific prevalence with no indication of the corresponding number of cases or the group sample size – for these it was assumed the overall age structure of the sample population was representative of the national average, and UNPDpopulation estimates closest to the study year were used to ascribe age-group sample sizes as proportions of the total number of participants[23].

Other studies provided the age-range for participants but no further age-specific breakdown or mean age, so the mean age between the sample's minimum and maximum ages was used. This was also applied for age-groups – for example a 65-69 age category would be treated as a sample with a mean age of 67. A small number of studies provided no mean age and no maximum age range. In these cases, a hypothetical maximum age of 80 was used to calculate the mean age.

During creation of the bubble graphs, mean age was plotted on the X-axis and prevalence per 1000 population on the Y-axis. Each study was not simply allocated a single plot point; instead, wherever it was possible, the mean age, sample size and age-specific prevalence of all individual age groups was recorded. This allowed wider coverage across the X-axis of the bubble graph rather than having all plots concentrated around the mean ages of 35-55. Bubble graphs take into account the size of each study and represent it through the plot's circle diameter, giving a visual representation of the sample size. Initially, three such bubble graphs were created – one using all the prevalence estimate data to get an overall picture of diabetes prevalence, and one each for males and females using sex-specific prevalence estimates. Later on several more graphs were created looking at the relationships between urban and rural residency and diabetes prevalence for both males and females.

Bubble graphs have the advantage that they take individual cohort size into account when calculating the trend line, and also can accommodate gaps in the data better than weighted mean box-plots. If using the latter and few studies are available for a particular age group, e.g. 50-59, then the particular plot for this age group may not be representative and can distort the overall trend. A bubble graph would take this lack of data into account but the trend line would not be distorted. In addition, the trend line equation of a bubble graph can be used to estimate the expected prevalence for any given age within the analysed age range as opposed to just the specific age group means. A weighted mean box-plot has the advantage that it can better display fluctuating disease trends, and would be preferable when investigating a disease for which there no established data on its relationship with age is available. However, bubble graphsare preferable over weighted mean box-plotswhen considering a disease such as diabetes, for which the steady prevalence increase with age has been previously well established [21].

III. Quality Assessment – FPG & OGTT combined studies

Authors	Country	Location	Urban/ Rural	Age Range	Study Size	Study Years	Sampling Methods	Biochemical Sample	Diagnostic Definition
Bhowmik et al (2012)	Bangladesh	Chandra	Rural	≥20	2293	2009	Cross-sectional study, randomised cluster sampling of villages	Venous plasma	FPG≥7.0mmol/L and/or 2hPG≥11.1mmol/L
Rahim et al (2008)	Bangladesh	Chandra	Rural	≥20	3954	2004	Cross-sectional study, randomised cluster sampling of villages	Capillary whole blood	FBG≥6.1mmol/L and/or 2hBG≥11.1mmol/L
Nazir et al (2012)	India	Chennai	Urban	≥20	Appendix	2003	Randomised multi-stage cluster sampling	Capillary plasma, venous plasma	FPG≥7.0mmol/L and/or 2hPG≥11.1mmol/L
Prasad et al (2012)	India	Odisha	Urban	20-80	1178	-	Multi-stage random sampling	Capillary plasma, venous plasma	FPG≥7.0mmol/L and/or 2hPG≥11.1mmol/L
Anjana et al (2011)	India	Tamil Nadu	Urban	≥20	1029	2009	Stratified multi-stage sampling in three states and one union territory	Capillary plasma	FPG≥7.0mmol/L and/or 2hPG≥12.2mmol/L
		Tamil Nadu	Rural	≥20	2480				
		Maharashtra	Urban	≥20	1093				
		Maharashtra	Rural	≥20	2476				
		Jharkhand	Urban	≥20	840				
		Jharkhand	Rural	≥20	2051				
		Chandigarh	Urban	≥20	839				
		Chandigarh	Rural	≥20	2247				
Deepa et al (2011)	India	Chennai	Urban	≥20	526	2008	Stratified randomised sampling	Capillary plasma, venous plasma	FPG≥7.0mmol/L and/or 2hPG≥11.1mmol/L
		Chennai	Urban	≥20	596				
Ravikumar et al (2011)	India	Chandigarh	Urban	≥20	2227	2008	Cross-sectional study, stratified systematic random sampling	Capillary plasma	FPG≥7.0mmol/L and/or 2hPG≥12.2mmol/L
Ramachandran et al (2008)	India	Chennai	Urban	≥20	2192	2006	Multi-stage stratified random sampling	Venous plasma	FPG≥7.0mmol/L and/or 2hPG≥11.1mmol/L
		Kanchipuram	Urban	≥20	2290				
		Panruti	Rural	≥20	2584				
Zargar et al (2008)	India	Kashmir Valley	Rural	20-40	3024	-	Multi-stage stratified random sampling	Capillary plasma, venous plasma	FPG≥7.0mmol/L and/or 2hPG≥11.1mmol/L
Sadikot et al (2004)	India	National	Urban	≥25	10617	1999-2002	Multi-stage randomised cluster sampling	Capillary plasma	FPG≥7.0mmol/L and/or 2hPG≥12.2mmol/L
		National	Rural	≥25	7746	1999-2002			

Ramachandran et al (2001)	India	National	Urban	≥20	11216	2000	Cluster random sampling	Capillary plasma	FPG≥7.0mmol/L and/or 2hPG≥12.2mmol/L
Hadaegh et al (2008)	Iran	Tehran	Urban	≥20	9489	1999-2001	Cross-sectional study, multistage cluster sampling	Venous plasma	FPG≥7.0mmol/L and/or 2hPG≥11.1mmol/L
Sadeghi et al (2007)	Iran	Arak, Isfahan, Najafabad	Both	≥19	12514	-	Multi-stage cluster random sampling	Venous plasma	FPG≥7.0mmol/L and/or 2hPG≥11.1mmol/L
Shrestha et al (2006)	Nepal	National	Urban	≥40	1012	2001-2002	Cluster sampling based on age reporting from a house-to-house census	Venous plasma	FPG≥7.0mmol/L and/or 2hPG≥11.1mmol/L
Katulanda et al (2008)	Sri Lanka	National	Both	≥18	4388	2005-2006	Cluster randomised sampling of households	Venous plasma	FPG≥7.0mmol/L and/or 2hPG≥11.1mmol/L

IV. Quality Assessment – FPG only studies

Authors	Country	Location	Urban/ Rural	Age Range	Study Size	Study Years	Sampling Methods	Biochemical Sample	Diagnostic Definition
Rahman et al (2007)	Bangladesh	Gazipur	Rural	≥20	975	2005	Multi-stage cluster sampling, with simple random selection of sample following census	Capillary whole blood	FBG≥6.1mmol/L
Hussain et al (2005)	Bangladesh	Chandra	Rural	≥20	4757	2005	Cross-sectional study, randomised cluster sampling of villages	Capillary whole blood	FBG≥6.1mmol/L
		Dhaka	Urban	≥20	1555				
Pandey et al (2013)	India	Haryana, Jaipur, Pune, Puducherry, Gandhigram	Rural	35-70	2616	2004-2007	Systematic stratified random sampling at each site	Venous plasma	FPG≥7.0mmol/L
		Jaipur, Kolkata, Kochi, Puducherry	Urban	35-70	2008				
Vaz et al (2011)	India	Goa	Rural	≥20	1266	-	Cross-sectional study, systematic random sampling	Venous plasma	FPG≥7.0mmol/L
Rao et al (2010)	India	Karnataka	Rural	≥30	1239	2006-2007	Cross-sectional community-based study, multistage stratified random sampling	Capillary whole blood	FBG≥6.1mmol/L
Vijayakumar et al (2009)	India	Kerala	Rural	≥18	1645	2007	Cross-sectional community-based study	Capillary plasma	FPG≥7.0mmol/L
Namperumalsamy et al (2009)	India	Theni	Both	≥30	25969	2005-2006	Multi-stage cluster sampling	Capillary plasma	FPG≥7.0mmol/L
Chow et al (2006)	India	Godavari	Rural	≥30	4538	2005	Cluster randomised sampling	Capillary plasma, venous plasma	FPG≥7.0mmol/L
Gupta et al (2003)	India	Jaipur	Both	≥20	1091	-	Stratified random sampling	Venous plasma	FPG≥7.0mmol/L
Misra et al (2001)	India	Gautum Nagar	Urban	≥18	532	1998	Random sampling based on electoral register	Venous plasma	FPG≥7.0mmol/L
Esteghamati et al (2009)	Iran	National	Both	25-64	3397	2007	Randomised cluster sampling of non-institutionalised individuals	Venous plasma	FPG≥7.0mmol/L
Azimi-Nezhad et	Iran	Khorosan	Both	20-64	3438	-	Randomised cluster	Venous plasma	FPG≥7.0mmol/L

al (2008)		province					sampling		
Sharma et al (2011)	Nepal	Eastern Nepal	Both	≥20	14008	-	Community-based door-to-door sampling	Venous plasma	FPG≥7.0mmol/L
Paudyal et al (2008)	Nepal	Kathmandu	Rural	≥40	1475	-	Community-based randomised cross-sectional study	Venous plasma	FPG≥7.0mmol/L
Basit et al (2011)	Pakistan	Southern Baluchistan	Rural	≥25	1264	2009	Cluster random sampling of households	Venous plasma	FPG≥7.0mmol/L
Zafar et al (2011)	Pakistan	Rawalpindi	Urban	12-80	1091	2008	Community-based random sampling of households	Venous plasma	FPG≥7.0mmol/L
Mahar et al (2010)	Pakistan	Gaddap Town, Karachi	Urban	30-90	19211	2007	Community-based randomised sampling	Venous plasma	FPG≥7.0mmol/L
Basit et al (2002)	Pakistan	Southern Baluchistan	Rural	≥25	2032	2002	Cluster random sampling of households	Venous plasma	FPG≥7.0mmol/L
Pinidiyapathirage et al (2013)	Sri Lanka	Ragama, Colombo	Urban	35-64	2986	2007	Cross-sectional study, randomised stratified sampling	Venous plasma	FPG≥7.0mmol/L
Wijewardene et al (2005)	Sri Lanka	Western province	Urban	30-65	4301	-	Multi-stage stratified cluster randomised sampling	Venous plasma	FPG≥7.0mmol/L
		North Central province	Rural	30-65	571				
		Southern province	Rural	30-65	331				
		Uva province	Rural	30-65	844				

V. Quality Assessment – OGTT only studies

Authors	Country	Location	Urban/ Rural	Age Range	Study Size	Study Years	Sampling Methods	Biochemical Sample	Diagnostic Definition
Boddula et al (2008)	India	Lucknow	Urban	≥30	1112	2003	Community-based randomised sampling	Venous plasma	2hPG≥11.1mmol/L
Ramachandran et al (2004)	India	Tamil Nadu	Rural	≥20	1213	2003	Community-based house-to-house sampling	Capillary whole blood	2hBG≥11.1mmol/L
Ramachandran et al (1994)	India	Chennai	Urban	≥60	873	-	Stratified random sampling	Capillary whole blood, venous plasma	2hPG≥11.1mmol/L
		Tamil Nadu	Rural	≥60	588				
Ramachandran et al (1992)	India	Chennai	Urban	≥20	900	-	Cluster randomised sampling	Capillary whole blood	2hBG≥11.1mmol/L
		Sriperumbudur	Rural	≥20	1038				

VI. Age-Specific Prevalence Data – Both Sexes Combined

Authors	Mean Age	Prevalence /1000 Population	Sample Size	Urban / Rural
Bhowmik et al (2012)	25	36	545	Rural
Bhowmik et al (2012)	35.5	77	704	Rural
Bhowmik et al (2012)	45.5	101	561	Rural
Bhowmik et al (2012)	65.5	104	481	Rural
Rahim et al (2008)	37.1	70	3954	Rural
Nazir et al (2012)	38.7	158	2188	Urban
Prasad et al (2012)	25	22	180	Urban
Prasad et al (2012)	35.5	66	287	Urban
Prasad et al (2012)	45.5	122	287	Urban
Prasad et al (2012)	55.5	288	240	Urban
Prasad et al (2012)	65.5	331	130	Urban
Prasad et al (2012)	75.5	278	54	Urban
Anjana et al (2011) i.	50	137	1029	Urban
Anjana et al (2011) ii.	50	78	2480	Rural
Anjana et al (2011) iii.	50	109	1093	Urban
Anjana et al (2011) iv.	50	65	2476	Rural
Anjana et al (2011) v.	50	135	840	Urban
Anjana et al (2011) vi.	50	30	2051	Rural
Anjana et al (2011) vii.	50	142	839	Urban
Anjana et al (2011) viii.	50	83	2247	Rural
Deepa et al (2011) i.	47.8	154	526	Urban
Deepa et al (2011) ii.	41.1	153	596	Urban
Ravikumar et al (2011)	42.7	157	2227	Urban
Zargar et al (2008)	30.8	25	3024	Rural
Sadikot et al (2004) i.	27	25	643	Urban
Sadikot et al (2004) i.	34.5	48	3012	Urban
Sadikot et al (2004) i.	44.5	63	3296	Urban
Sadikot et al (2004) i.	54.5	67	2948	Urban
Sadikot et al (2004) i.	70	82	718	Urban
Sadikot et al (2004) ii.	27	17	544	Rural
Sadikot et al (2004) ii.	34.5	19	2317	Rural
Sadikot et al (2004) ii.	44.5	29	2348	Rural
Sadikot et al (2004) ii.	54.5	27	1962	Rural
Sadikot et al (2004) ii.	70	71	575	Rural
Ramachandran et al (2001)	24.5	24	2425	Urban
Ramachandran et al (2001)	34.5	70	2908	Urban
Ramachandran et al (2001)	44.5	165	2571	Urban
Ramachandran et al (2001)	54.5	263	1692	Urban
Ramachandran et al (2001)	64.5	291	1199	Urban
Ramachandran et al (2001)	75	259	533	Urban
Hadaegh et al (2008)	24.5	16	1865	Urban

Hadaegh et al (2008)	34.5	48	2518	Urban
Hadaegh et al (2008)	44.5	142	1906	Urban
Hadaegh et al (2008)	54.5	251	1531	Urban
Hadaegh et al (2008)	64.5	321	1301	Urban
Hadaegh et al (2008)	75	338	368	Urban
Sadeghi et al (2007)	21.5	7	2690	Both
Sadeghi et al (2007)	29.5	16	3616	Both
Sadeghi et al (2007)	39.5	55	2602	Both
Sadeghi et al (2007)	49.5	109	1789	Both
Sadeghi et al (2007)	59.5	164	926	Both
Sadeghi et al (2007)	72.5	188	901	Both
Shrestha et al (2006)	44.5	124	443	Urban
Shrestha et al (2006)	54.5	227	255	Urban
Shrestha et al (2006)	67	256	250	Urban
Shrestha et al (2006)	77.5	250	64	Urban
Katulanda et al (2008)	24.5	13	643	Both
Katulanda et al (2008)	34.5	64	887	Both
Katulanda et al (2008)	44.5	124	1090	Both
Katulanda et al (2008)	54.5	174	896	Both
Katulanda et al (2008)	64.5	212	537	Both
Katulanda et al (2008)	75	235	335	Both
Rahman et al (2007)	25	28	360	Rural
Rahman et al (2007)	35.5	85	271	Rural
Rahman et al (2007)	45.5	81	142	Rural
Rahman et al (2007)	65.5	188	202	Rural
Hussain et al (2005) i.	25	15	2080	Rural
Hussain et al (2005) i.	35.5	22	1252	Rural
Hussain et al (2005) i.	45.5	29	655	Rural
Hussain et al (2005) i.	65.5	39	770	Rural
Hussain et al (2005) ii.	25	43	819	Urban
Hussain et al (2005) ii.	35.5	96	395	Urban
Hussain et al (2005) ii.	45.5	147	217	Urban
Hussain et al (2005) ii.	65.5	169	124	Urban
Pandey et al (2013) i.	46.7	43	2616	Rural
Pandey et al (2013) ii.	48.4	151	2008	Urban
Vaz et al (2011)	29.5	24	841	Rural
Vaz et al (2011)	49.5	183	262	Rural
Vaz et al (2011)	69.5	372	148	Rural
Rao et al (2010)	51.3	160	1239	Rural
Vijayakumar et al (2009)	23.5	7	297	Rural
Vijayakumar et al (2009)	37	77	441	Rural
Vijayakumar et al (2009)	52	216	408	Rural
Vijayakumar et al (2009)	70	282	415	Rural
Namperumalsamy et al (2009)	32.5	75	14181	Both
Namperumalsamy et al (2009)	62.5	147	11788	Both
Chow et al (2006)	34.5	52	1638	Rural

Chow et al (2006)	44.5	135	1271	Rural
Chow et al (2006)	54.5	191	876	Rural
Chow et al (2006)	70	205	753	Rural
Gupta et al (2003)	24.5	11	187	Both
Gupta et al (2003)	34.5	52	290	Both
Gupta et al (2003)	44.5	92	238	Both
Gupta et al (2003)	54.5	261	211	Both
Gupta et al (2003)	70	242	165	Both
Misra et al (2001)	35.4	103	532	Urban
Esteghamati et al (2009)	29.5	32	843	Both
Esteghamati et al (2009)	39.5	92	902	Both
Esteghamati et al (2009)	49.5	140	869	Both
Esteghamati et al (2009)	59.5	188	783	Both
Azimi-Nezhad et al (2008)	24.5	99	506	Both
Azimi-Nezhad et al (2008)	34.5	189	898	Both
Azimi-Nezhad et al (2008)	44.5	55	993	Both
Azimi-Nezhad et al (2008)	54.5	85	827	Both
Azimi-Nezhad et al (2008)	70	109	504	Both
Sharma et al (2011)	30	19	7519	Both
Sharma et al (2011)	50.5	102	4727	Both
Sharma et al (2011)	70.5	154	1664	Both
Paudyal et al (2008)	44.5	30	575	Rural
Paudyal et al (2008)	54.5	39	410	Rural
Paudyal et al (2008)	64.5	55	271	Rural
Paudyal et al (2008)	75	41	219	Rural
Basit et al (2011)	42.3	142	1264	Rural
Zafar et al (2011)	38	127	566	Urban
Zafar et al (2011)	63	393	150	Urban
Zafar et al (2011)	78	318	22	Urban
Mahar et al (2010)	35	38	11204	Urban
Mahar et al (2010)	45.5	148	4530	Urban
Mahar et al (2010)	55.5	182	2213	Urban
Mahar et al (2010)	65.5	160	999	Urban
Mahar et al (2010)	75.5	83	265	Urban
Basit et al (2002)	38.9	72	2032	Rural
Pinidiyapathirage et al (2013)	39.5	124	515	Urban
Pinidiyapathirage et al (2013)	49.5	231	1140	Urban
Pinidiyapathirage et al (2013)	59.5	308	1330	Urban
Wijewardene et al (2005) i.	32.5	111	570	Urban
Wijewardene et al (2005) i.	40.5	149	1431	Urban
Wijewardene et al (2005) i.	50.5	193	1397	Urban
Wijewardene et al (2005) i.	60.5	226	903	Urban
Wijewardene et al (2005) ii.	32.5	21	94	Rural
Wijewardene et al (2005) ii.	40.5	68	235	Rural
Wijewardene et al (2005) ii.	50.5	69	160	Rural
Wijewardene et al (2005) ii.	60.5	134	82	Rural

Wijewardene et al (2005) iii.	40.5	61	114	Rural
Wijewardene et al (2005) iii.	50.5	42	119	Rural
Wijewardene et al (2005) iv.	32.5	31	131	Rural
Wijewardene et al (2005) iv.	40.5	67	315	Rural
Wijewardene et al (2005) iv.	50.5	77	248	Rural
Wijewardene et al (2005) iv.	60.5	107	150	Rural
Boddula et al (2008)	55	246	1112	Urban
Ramachandran et al (2008) i.	38.2	186	2192	Urban
Ramachandran et al (2008) ii.	36.8	164	2290	Urban
Ramachandran et al (2008) iii.	38	92	2584	Rural
Ramachandran et al (2004)	41	63	1213	Rural
Ramachandran et al (1994) i.	70	237	873	Urban
Ramachandran et al (1994) ii.	70	99	588	Rural
Ramachandran et al (1992)	29.5	8	253	Urban
Ramachandran et al (1992)	39.5	85	331	Urban
Ramachandran et al (1992)	49.5	168	149	Urban
Ramachandran et al (1992)	59.5	200	45	Urban
Ramachandran et al (1992)	72.5	273	33	Urban

VII. Age-Specific Prevalence Data – Males

Authors	Mean Age	Prevalence / 1000 Population	Sample Size	Urban / Rural
Bhowmik et al (2012)	25	36	167	Rural
Bhowmik et al (2012)	35.5	95	231	Rural
Bhowmik et al (2012)	45.5	103	223	Rural
Bhowmik et al (2012)	65.5	131	221	Rural
Rahim et al (2008)	39	75	1592	Rural
Prasad et al (2012)	25	24	83	Urban
Prasad et al (2012)	35.5	78	115	Urban
Prasad et al (2012)	45.5	109	138	Urban
Prasad et al (2012)	55.5	304	138	Urban
Prasad et al (2012)	65.5	338	77	Urban
Prasad et al (2012)	75.5	282	39	Urban
Sadikot et al (2004) i.	44.5	56	5379	Urban
Sadikot et al (2004) ii.	44.1	25	3629	Rural
Ramachandran et al (2001)	24.5	23	1242	Urban
Ramachandran et al (2001)	34.5	73	1237	Urban
Ramachandran et al (2001)	44.5	160	1214	Urban
Ramachandran et al (2001)	54.5	252	793	Urban
Ramachandran et al (2001)	64.5	311	531	Urban
Ramachandran et al (2001)	75	263	271	Urban
Hadaegh et al (2008)	24.5	19	694	Urban
Hadaegh et al (2008)	34.5	44	1054	Urban
Hadaegh et al (2008)	44.5	121	775	Urban
Hadaegh et al (2008)	54.5	240	605	Urban
Hadaegh et al (2008)	64.5	289	637	Urban
Hadaegh et al (2008)	75	340	241	Urban
Sadeghi et al (2007)	39	54	6123	Both
Katulanda et al (2008)	24.5	8	260	Both
Katulanda et al (2008)	34.5	77	333	Both
Katulanda et al (2008)	44.5	111	418	Both
Katulanda et al (2008)	54.5	174	347	Both
Katulanda et al (2008)	64.5	181	212	Both
Katulanda et al (2008)	75	229	150	Both
Rahman et al (2007)	25	28	108	Rural
Rahman et al (2007)	35.5	85	94	Rural
Rahman et al (2007)	45.5	81	62	Rural
Rahman et al (2007)	65.5	188	96	Rural
Hussain et al (2005) i.	25	11	700	Rural
Hussain et al (2005) i.	35.5	17	589	Rural
Hussain et al (2005) i.	45.5	26	337	Rural
Hussain et al (2005) i.	65.5	29	411	Rural
Hussain et al (2005) ii.	25	30	330	Urban

Hussain et al (2005) ii.	35.5	98	194	Urban
Hussain et al (2005) ii.	45.5	105	124	Urban
Hussain et al (2005) ii.	65.5	169	83	Urban
Vaz et al (2011)	39	84	609	Rural
Vijayakumar et al (2009)	48.2	165	624	Rural
Gupta et al (2003)	24.5	20	98	Both
Gupta et al (2003)	34.5	61	147	Both
Gupta et al (2003)	44.5	126	111	Both
Gupta et al (2003)	54.5	265	98	Both
Gupta et al (2003)	70	244	78	Both
Misra et al (2001)	37.8	112	170	Urban
Sharma et al (2011)	41.4	81	5326	Both
Basit et al (2011)	39.5	123	168	Rural
Basit et al (2011)	49.5	203	121	Rural
Basit et al (2011)	59.5	382	76	Rural
Basit et al (2011)	72.5	315	59	Rural
Basit et al (2002)	39.5	78	268	Rural
Basit et al (2002)	49.5	182	178	Rural
Basit et al (2002)	59.5	295	129	Rural
Basit et al (2002)	72.5	111	95	Rural
Boddula et al (2008)	55	284	557	Urban
Ramachandran et al (2008) i.	38.2	209	1053	Urban
Ramachandran et al (2008) ii.	36.8	171	988	Urban
Ramachandran et al (2008) iii.	38	104	1280	Rural
Ramachandran et al (2004)	41	74	497	Rural
Ramachandran et al (1992)	29.5	11	88	Urban
Ramachandran et al (1992)	39.5	105	190	Urban
Ramachandran et al (1992)	49.5	185	108	Urban
Ramachandran et al (1992)	59.5	118	17	Urban
Ramachandran et al (1992)	72.5	286	14	Urban
Shrestha et al (2006)	44.5	159	188	Urban
Shrestha et al (2006)	54.5	327	98	Urban
Shrestha et al (2006)	67	321	106	Urban
Shrestha et al (2006)	77.5	258	31	Urban
Rao et al (2010)	50	188	434	Rural
Zafar et al (2011)	50	154	293	Urban
Pinidiyapathirage et al (2013)	39.5	158	241	Urban
Pinidiyapathirage et al (2013)	49.5	212	501	Urban
Pinidiyapathirage et al (2013)	59.5	280	607	Urban
Wijewardene et al (2005) i.	32.5	120	285	Urban
Wijewardene et al (2005) i.	40.5	166	647	Urban
Wijewardene et al (2005) i.	50.5	216	562	Urban
Wijewardene et al (2005) i.	60.5	211	397	Urban
Wijewardene et al (2005) ii.	32.5	22	45	Rural
Wijewardene et al (2005) ii.	40.5	78	115	Rural

Wijewardene et al (2005) ii.	50.5	86	70	Rural
Wijewardene et al (2005) ii.	60.5	89	45	Rural
Wijewardene et al (2005) iii.	40.5	82	49	Rural
Wijewardene et al (2005) iii.	50.5	64	47	Rural
Wijewardene et al (2005) iv.	32.5	45	70	Rural
Wijewardene et al (2005) iv.	40.5	57	138	Rural
Wijewardene et al (2005) iv.	50.5	104	97	Rural
Wijewardene et al (2005) iv.	60.5	73	82	Rural

VIII. Age-Specific Prevalence Data – Females

Authors	Mean Age	Prevalence / 1000 Population	Sample Size	Urban / Rural
Bhowmik et al (2012)	25	37	378	Rural
Bhowmik et al (2012)	35.5	68	473	Rural
Bhowmik et al (2012)	45.5	101	338	Rural
Bhowmik et al (2012)	65.5	81	260	Rural
Rahim et al (2008)	35.9	67	2375	Rural
Prasad et al (2012)	25	21	97	Urban
Prasad et al (2012)	35.5	58	172	Urban
Prasad et al (2012)	45.5	134	149	Urban
Prasad et al (2012)	55.5	265	102	Urban
Prasad et al (2012)	65.5	321	53	Urban
Prasad et al (2012)	75.5	267	15	Urban
Sadikot et al (2004) i.	45.1	58	5238	Urban
Sadikot et al (2004) ii.	44.3	25	4117	Rural
Ramachandran et al (2001)	24.5	24	1183	Urban
Ramachandran et al (2001)	34.5	68	1671	Urban
Ramachandran et al (2001)	44.5	169	1357	Urban
Ramachandran et al (2001)	54.5	273	899	Urban
Ramachandran et al (2001)	64.5	276	668	Urban
Ramachandran et al (2001)	75	255	262	Urban
Hadaegh et al (2008)	24.5	14	1171	Urban
Hadaegh et al (2008)	34.5	51	1464	Urban
Hadaegh et al (2008)	44.5	156	1131	Urban
Hadaegh et al (2008)	54.5	259	926	Urban
Hadaegh et al (2008)	64.5	352	664	Urban
Hadaegh et al (2008)	75	333	127	Urban
Sadeghi et al (2007)	39	71	6391	Both
Katulanda et al (2008)	24.5	18	383	Both
Katulanda et al (2008)	34.5	51	554	Both
Katulanda et al (2008)	44.5	140	672	Both
Katulanda et al (2008)	54.5	174	549	Both
Katulanda et al (2008)	64.5	242	325	Both
Katulanda et al (2008)	75	240	185	Both
Rahman et al (2007)	25	36	252	Rural
Rahman et al (2007)	35.5	130	177	Rural
Rahman et al (2007)	45.5	75	80	Rural
Rahman et al (2007)	65.5	104	106	Rural
Hussain et al (2005) i.	25	17	1380	Rural
Hussain et al (2005) i.	35.5	25	663	Rural
Hussain et al (2005) i.	45.5	31	318	Rural
Hussain et al (2005) i.	65.5	50	359	Rural
Hussain et al (2005) ii.	25	51	489	Urban

Hussain et al (2005) ii.	35.5	94	201	Urban
Hussain et al (2005) ii.	45.5	204	93	Urban
Hussain et al (2005) ii.	65.5	170	41	Urban
Vaz et al (2011)	39	120	657	Rural
Vijayakumar et al (2009)	46.2	135	1021	Rural
Gupta et al (2003)	34.5	42	143	Both
Gupta et al (2003)	44.5	63	127	Both
Gupta et al (2003)	54.5	257	113	Both
Gupta et al (2003)	70	241	87	Both
Misra et al (2001)	34.3	99	362	Urban
Sharma et al (2011)	41.4	53	8682	Both
Basit et al (2011)	39.5	77	335	Rural
Basit et al (2011)	49.5	262	242	Rural
Basit et al (2011)	59.5	240	150	Rural
Basit et al (2011)	72.5	377	113	Rural
Basit et al (2002)	39.5	45	563	Rural
Basit et al (2002)	49.5	53	366	Rural
Basit et al (2002)	59.5	188	252	Rural
Basit et al (2002)	72.5	75	181	Rural
Boddula et al (2008)	55	207	555	Urban
Ramachandran et al (2008) i.	38.2	167	1139	Urban
Ramachandran et al (2008) ii.	36.8	159	1302	Urban
Ramachandran et al (2008) iii.	38	80	1304	Rural
Ramachandran et al (2004)	40.5	56	716	Rural
Ramachandran et al (1992)	22	20	49	Urban
Ramachandran et al (1992)	29.5	6	165	Urban
Ramachandran et al (1992)	39.5	57	141	Urban
Ramachandran et al (1992)	49.5	122	41	Urban
Ramachandran et al (1992)	59.5	250	28	Urban
Ramachandran et al (1992)	72.5	263	19	Urban
Shrestha et al (2006)	44.5	98	255	Urban
Shrestha et al (2006)	54.5	165	157	Urban
Shrestha et al (2006)	67	208	144	Urban
Shrestha et al (2006)	77.5	243	33	Urban
Pandey et al (2013) i.	46.7	43	2616	Rural
Pandey et al (2013) ii.	48.4	151	2008	Urban
Rao et al (2010)	52	144	805	Rural
Zafar et al (2011)	50	123	798	Urban
Pinidiyapathirage et al (2013)	39.5	95	274	Urban
Pinidiyapathirage et al (2013)	49.5	246	639	Urban
Pinidiyapathirage et al (2013)	59.5	331	723	Urban
Wijewardene et al (2005) i.	32.5	101	285	Urban
Wijewardene et al (2005) i.	40.5	135	784	Urban
Wijewardene et al (2005) i.	50.5	178	835	Urban
Wijewardene et al (2005) i.	60.5	238	506	Urban

Wijewardene et al (2005) ii.	32.5	20	49	Rural
Wijewardene et al (2005) ii.	40.5	58	120	Rural
Wijewardene et al (2005) ii.	50.5	56	90	Rural
Wijewardene et al (2005) ii.	60.5	179	37	Rural
Wijewardene et al (2005) iii.	40.5	46	65	Rural
Wijewardene et al (2005) iii.	50.5	28	72	Rural
Wijewardene et al (2005) iv.	32.5	17	61	Rural
Wijewardene et al (2005) iv.	40.5	74	177	Rural
Wijewardene et al (2005) iv.	50.5	59	151	Rural
Wijewardene et al (2005) iv.	60.5	145	68	Rural

IX. Sex-specific Bubble Graphs for Residency

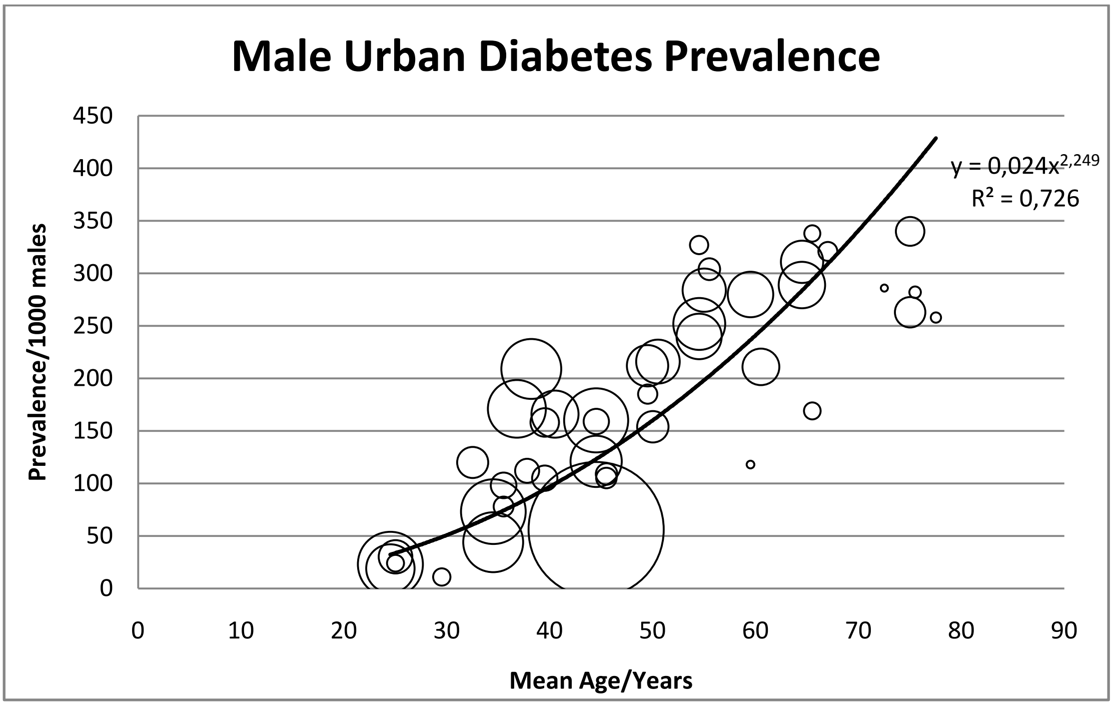

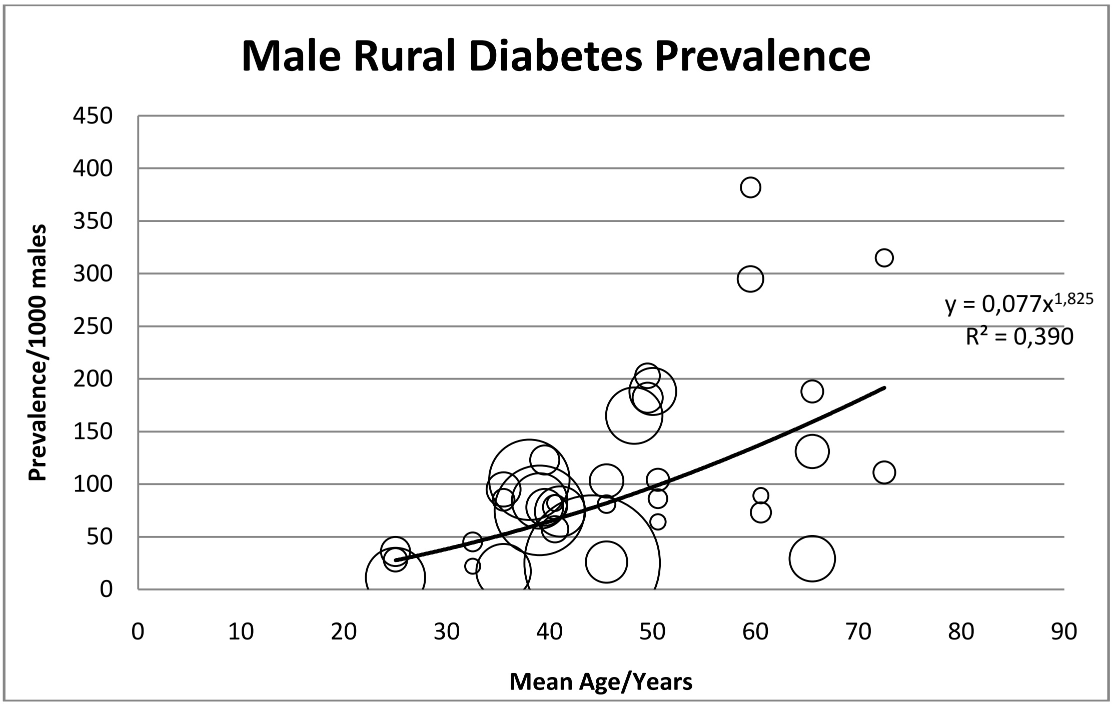

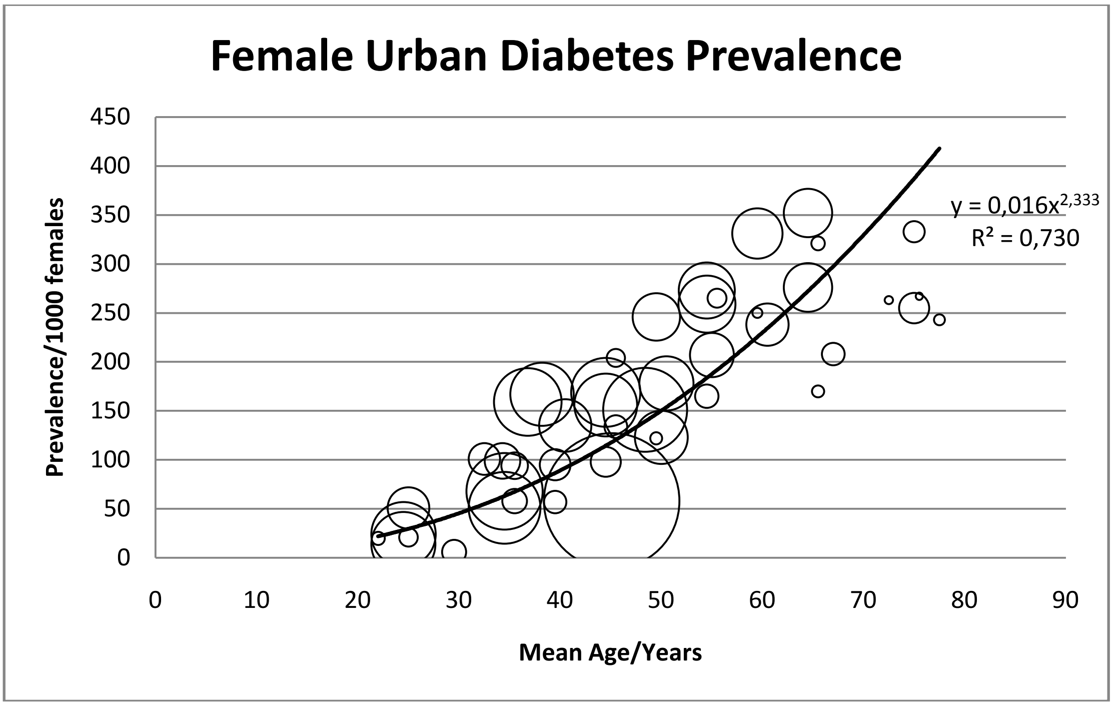
Female Urban Diabetes Prevalence
450
400
350
300
250
200
150
100
50
0
Prevalence/1000 females
y = 0,016x2,333
R² = 0,730
0
10
20
30
40
50
60
70
80
90
Mean Age/Years

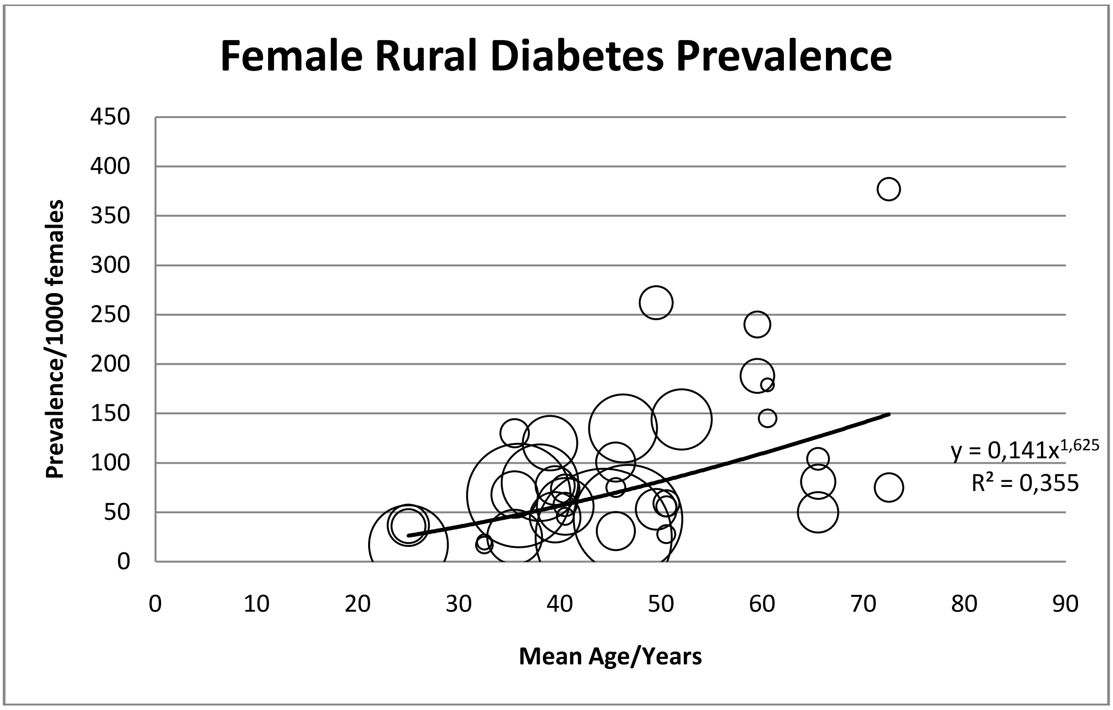
Female Rural Diabetes Prevalence
450
400
350
300
250
200
150
100
50
0
Prevalence/1000 females
y = 0,141x1,625
R² = 0,355
0
10
20
30
40
50
60
70
80
90
Mean Age/Years
